# Supplementary material for: Cytosolic retention of HtrA2 during mitochondrial protein import stress triggers the DELE1-HRI pathway
Source: Commun Biol. 2024 Mar 30;7:391. doi: 10.1038/s42003-024-06107-7 (PMC10981713; doi:10.1038/s42003-024-06107-7)
Supplement: Supplementary file 2 — Supplementary Information [file 42003_2024_6107_MOESM2_ESM.pdf]

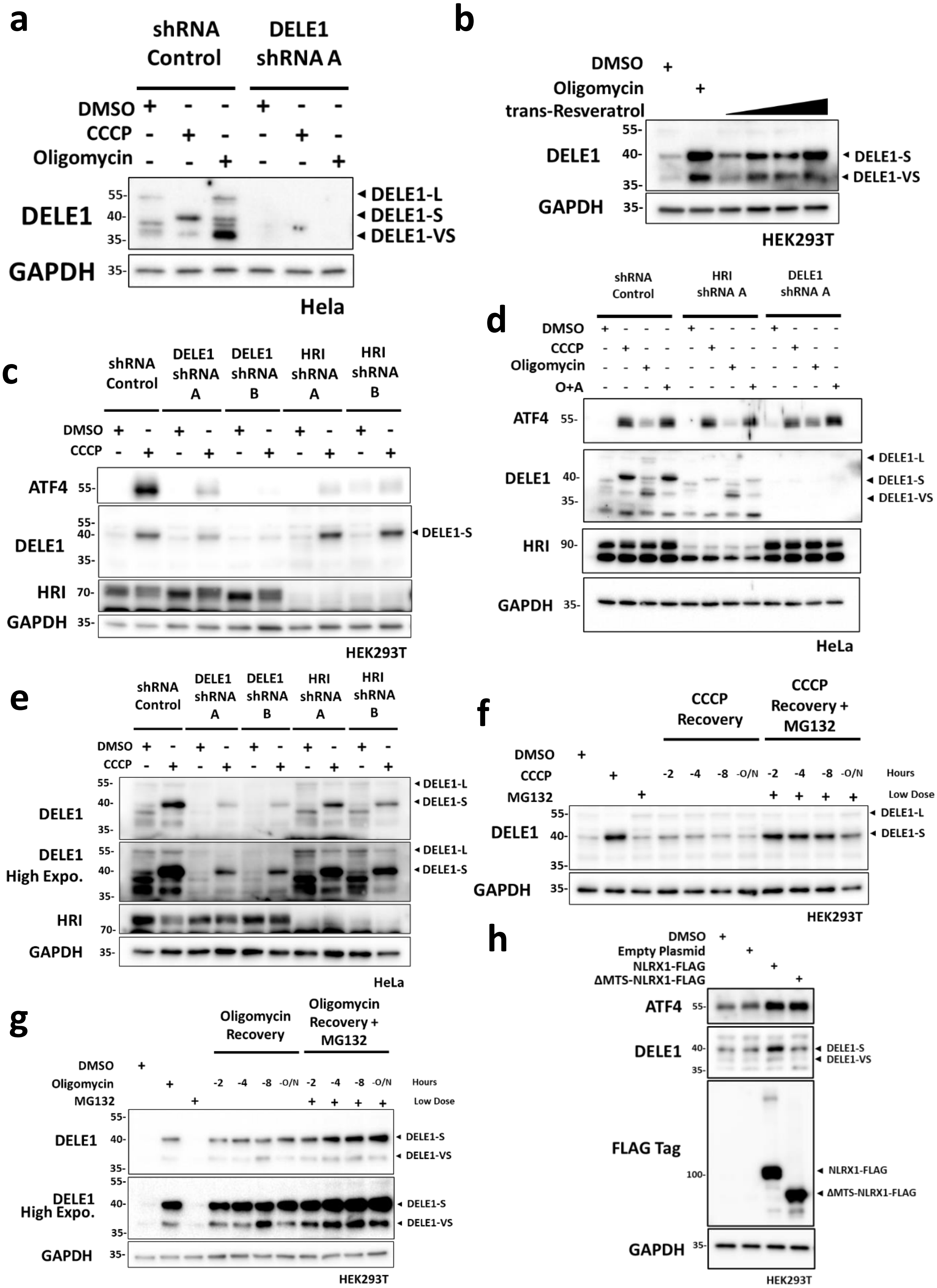
a
shRNA Control
DELE1 shRNA A
DMSO
CCCP
Oligomycin
DELE1
GAPDH
DELE1-L
DELE1-S
DELE1-VS
Hela
b
DMSO
Oligomycin
trans-Resveratrol
DELE1
GAPDH
DELE1-S
DELE1-VS
HEK293T
c
shRNA Control
DELE1 shRNA A
DELE1 shRNA B
HRI shRNA A
HRI shRNA B
ATF4
DELE1
HRI
GAPDH
DELE1-S
HEK293T
d
HRI shRNA A
O+A
HeLa
e
DELE1 High Expo.
f
CCCP Recovery
CCCP Recovery + MG132
MG132
Hours
Low Dose
g
Oligomycin Recovery
Oligomycin Recovery + MG132
h
Empty Plasmid
NLRX1-FLAG
ΔMTS-NLRX1-FLAG
FLAG Tag

Supplementary Fig 1. The DELE1-HRI pathway activates the ISR in response to MPIS.

a SC and shDELE1 A HeLa cells were treated with DMSO, CCCP (20 μM) or oligomycin (10 μM) for 4 hours, and whole-cell lysates were analyzed by WB. **b** WT HEK293T cells were treated with oligomycin (10 μM) and an increasing dose of trans-Resveratrol (6.25-50 μM) for 4 hours, and whole-cell lysates were analyzed by WB. **c** SC, shHRI A, shHRI B, shDELE1 A and shDELE1 B HEK293T cells were treated with DMSO or CCCP (20 μM) for 4 hours and whole-cell lysates were analyzed by WB. **d** SC, shHRI A and shDELE1 A HeLa cells were treated with DMSO, CCCP (20 μM), oligomycin (10 μM) and oligomycin + antimycin (O+A, 10 μM and 1 μM respectively) for 4 hours and whole-cell lysates were analyzed by WB. **e** SC, shHRI A, shHRI B, shDELE1 A and shDELE1 B HeLa cells were treated with DMSO or CCCP (20 μM) for 4 hours and whole-cell lysates were analyzed by WB. **f** WT HEK293T cells were first treated with CCCP (20 μM) for 4 hours, and then allow to recover in normal media for 2, 4, 8 hours and overnight, with or without the addition of a low dose of MG-132 (1 μM). An overnight treatment of MG-132 (1 μM) was included as control. Whole-cell lysates were analyzed by WB. **g** WT HEK293T cells were first treated with oligomycin (10 μM) for 4 hours, and then allow to recover in normal media for 2, 4, 8 hours and overnight, with or without the addition of a low dose of MG-132 (1 μM). An overnight treatment of MG-132 (1 μM) was included as control. Whole-cell lysates were analyzed by WB. **h** WT HEK293T cells were transfected with 0.4μg of empty plasmid, OE plasmid of NLRX1-FLAG or OE plasmid of ΔMTS-NLEX1-FLAG for 2 days and whole-cell lysates were analyzed by WB.

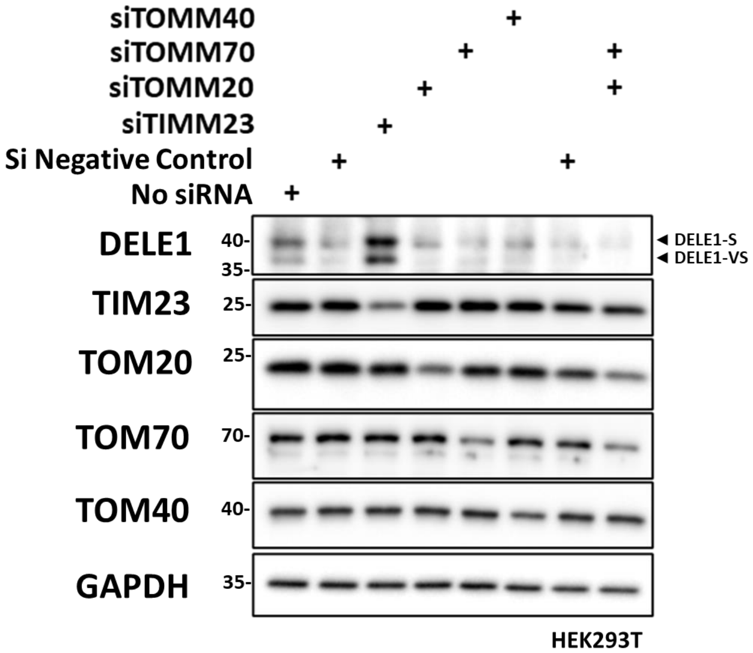
siTOMM40
siTOMM70
siTOMM20
siTIMM23
Si Negative Control
No siRNA
DELE1
40-
35-
DELE1-S
DELE1-VS
TIM23
25-
TOM20
25-
TOM70
70-
TOM40
40-
GAPDH
35-
HEK293T

Supplementary Fig 2. Inhibition of the TIM23 complex activates DELE1 signaling.

WT HEK293T cells were transfected with no siRNA or the corresponding siRNA for 2 days and whole-cell lysates were analyzed by WB.

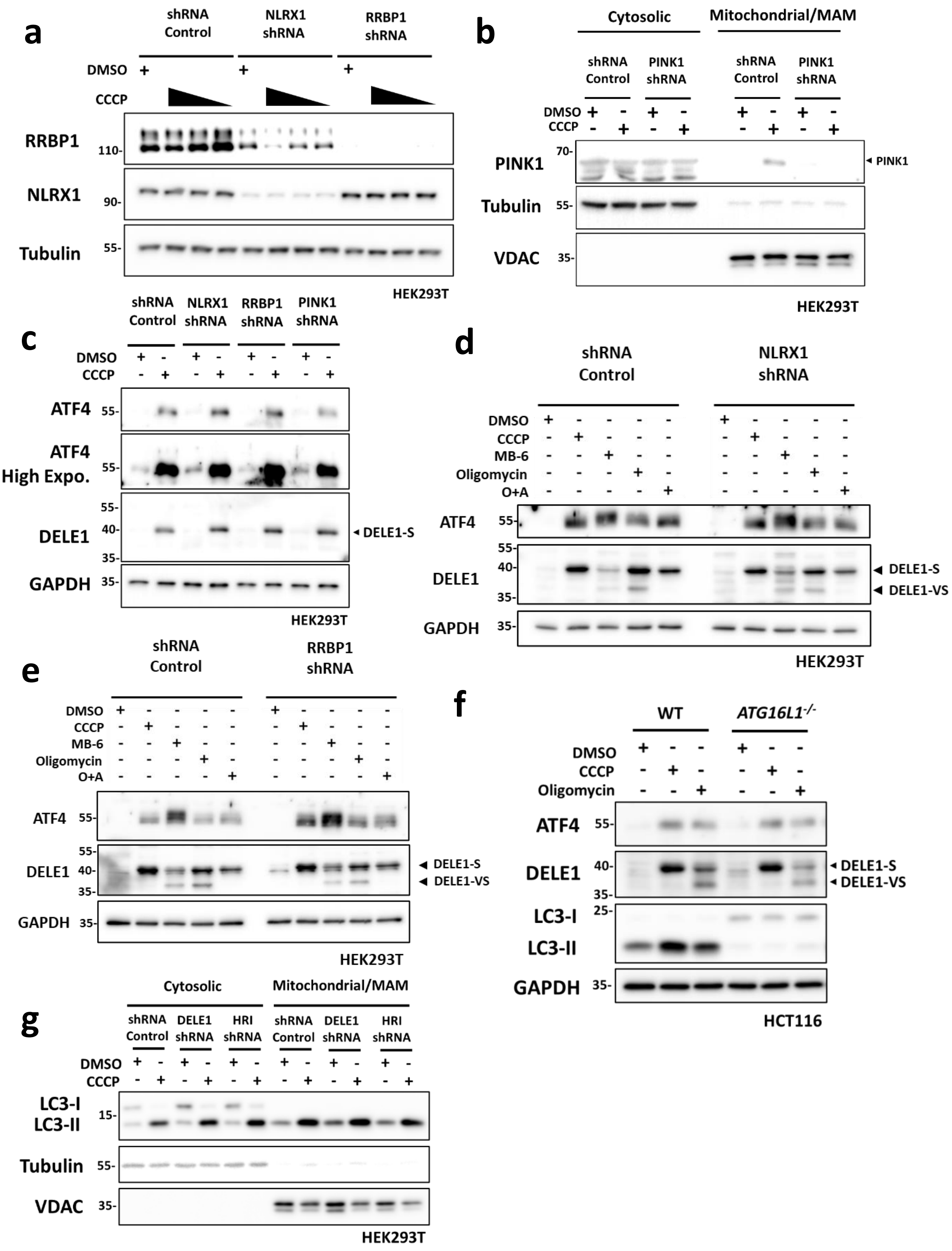
a
shRNA Control
NLRX1 shRNA
RRBP1 shRNA
DMSO
CCCP
RRBP1
110-
NLRX1
90-
Tubulin
55-
HEK293T
b
Cytosolic
Mitochondrial/MAM
shRNA Control
PINK1 shRNA
DMSO
CCCP
PINK1
70-
Tubulin
55-
VDAC
35-
HEK293T
c
shRNA Control
NLRX1 shRNA
RRBP1 shRNA
PINK1 shRNA
ATF4
ATF4 High Expo.
DELE1
DELE1-S
GAPDH
HEK293T
d
shRNA Control
NLRX1 shRNA
MB-6
Oligomycin
O+A
DELE1-VS
HEK293T
e
shRNA Control
RRBP1 shRNA
HEK293T
f
WT
ATG16L1-/-
LC3-I
LC3-II
HCT116
g
Cytosolic
Mitochondrial/MAM
shRNA Control
DELE1 shRNA
HRI shRNA
HEK293T

Supplementary Fig 3. The DELE1-HRI mediated ISR senses and responds to MPIS independently from mitophagy.

a SC, shNLRX1 and shRRBP1 HEK293T cells were treated with DMSO or increasing doses of CCCP (up to 20 μM) for 20 hours and whole-cell lysates were analyzed by WB. **b** Mitochondrial fractionation of SC and shPINK1 HEK293T cells lysates following DMSO or CCCP (20 μM) for 20 hours and analyzed by WB. **c** SC and shNLRX1, shRRBP1 and shPINK1 HEK293T cells were treated with DMSO or CCCP (20 μM) for 20 hours and whole-cell lysates were analyzed by WB. **d** SC and shNLRX1 HEK293T cells with DMSO or the same panel of mitochondrial stress inducers as in (Fig. 1c) for 4 hours and whole-cell lysates were analyzed by Western WB. **e** SC and shRRBP1 HEK293T cells with DMSO or the same panel of mitochondrial stress inducers as in (Fig. 1c) for 4 hours and whole-cell lysates were analyzed by Western WB. **f** WT and ATG16L1 knockout HCT116 cells treated with DMSO, CCCP (20 μM) or oligomycin (10 μM) for 4 hours and whole-cell lysates were analyzed by WB. **g** Mitochondrial fractionation of SC, shHRI and shDELE1 HEK293T cells lysates following DMSO or CCCP (20 μM) for 20 hours and analyzed by WB.

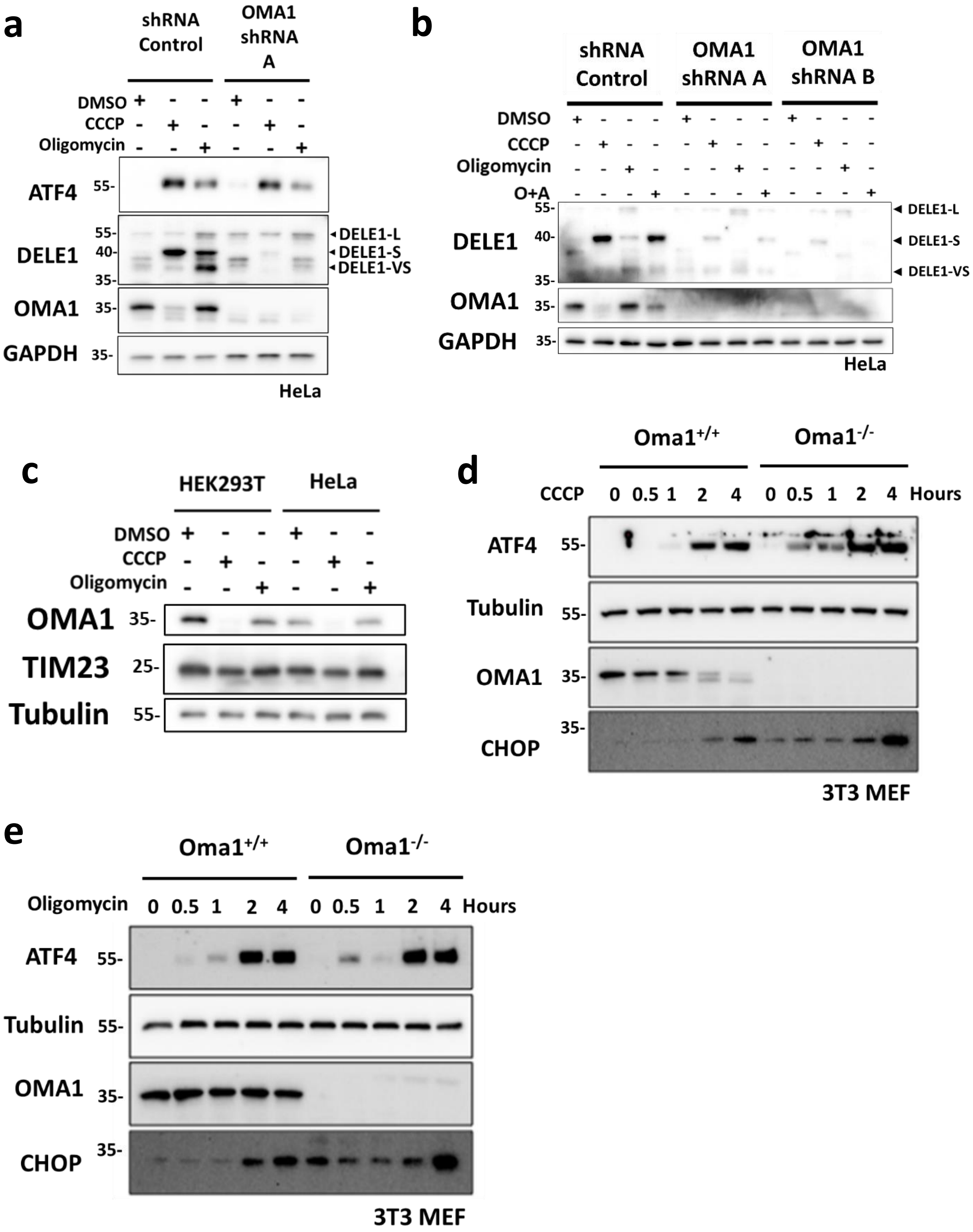
a
shRNA Control
OMA1 shRNA A
DMSO
CCCP
Oligomycin
ATF4
DELE1
OMA1
GAPDH
DELE1-L
DELE1-S
DELE1-VS
HeLa
b
shRNA Control
OMA1 shRNA A
OMA1 shRNA B
DMSO
CCCP
Oligomycin
O+A
DELE1
OMA1
GAPDH
DELE1-L
DELE1-S
DELE1-VS
HeLa
c
HEK293T
HeLa
DMSO
CCCP
Oligomycin
OMA1
TIM23
Tubulin
d
Oma1+/+
Oma1-/-
CCCP 0 0.5 1 2 4 0 0.5 1 2 4 Hours
ATF4
Tubulin
OMA1
CHOP
3T3 MEF
e
Oma1+/+
Oma1-/-
Oligomycin 0 0.5 1 2 4 0 0.5 1 2 4 Hours
ATF4
Tubulin
OMA1
CHOP
3T3 MEF

Supplementary Fig 4. Cell type-dependent cleavage of DELE1 by OMA1 following MPIS

a SC and shOMA1 A HeLa cells were treated with DMSO, CCCP (20 μM) and oligomycin (10 μM) to 4 hours and whole-cell lysates were analyzed by WB. **b** SC, shOMA1 A and shOMA1 B HeLa cells were treated with DMSO, CCCP (20 μM), oligomycin (10 μM) or O+A (10 μM and 1 μM) for 4 hours and whole-cell lysates were analyzed by WB. **c** WT HEK293T and HeLa cells were treated with DMSO, CCCP (20 μM) and oligomycin (10 μM) to 4 hours and whole-cell lysates were analyzed by WB. **d** WT ($Oma1^{+/+}$) and $Oma1^{-/-}$ 3T3 MEF cells were treated with 20 μM CCCP for the indicated time and whole-cell lysates were analyzed by WB. **e** WT ($Oma1^{+/+}$) and $Oma1^{-/-}$ 3T3 MEF cells were treated with 10 μM oligomycin for the indicated time and whole-cell lysates were analyzed by WB.

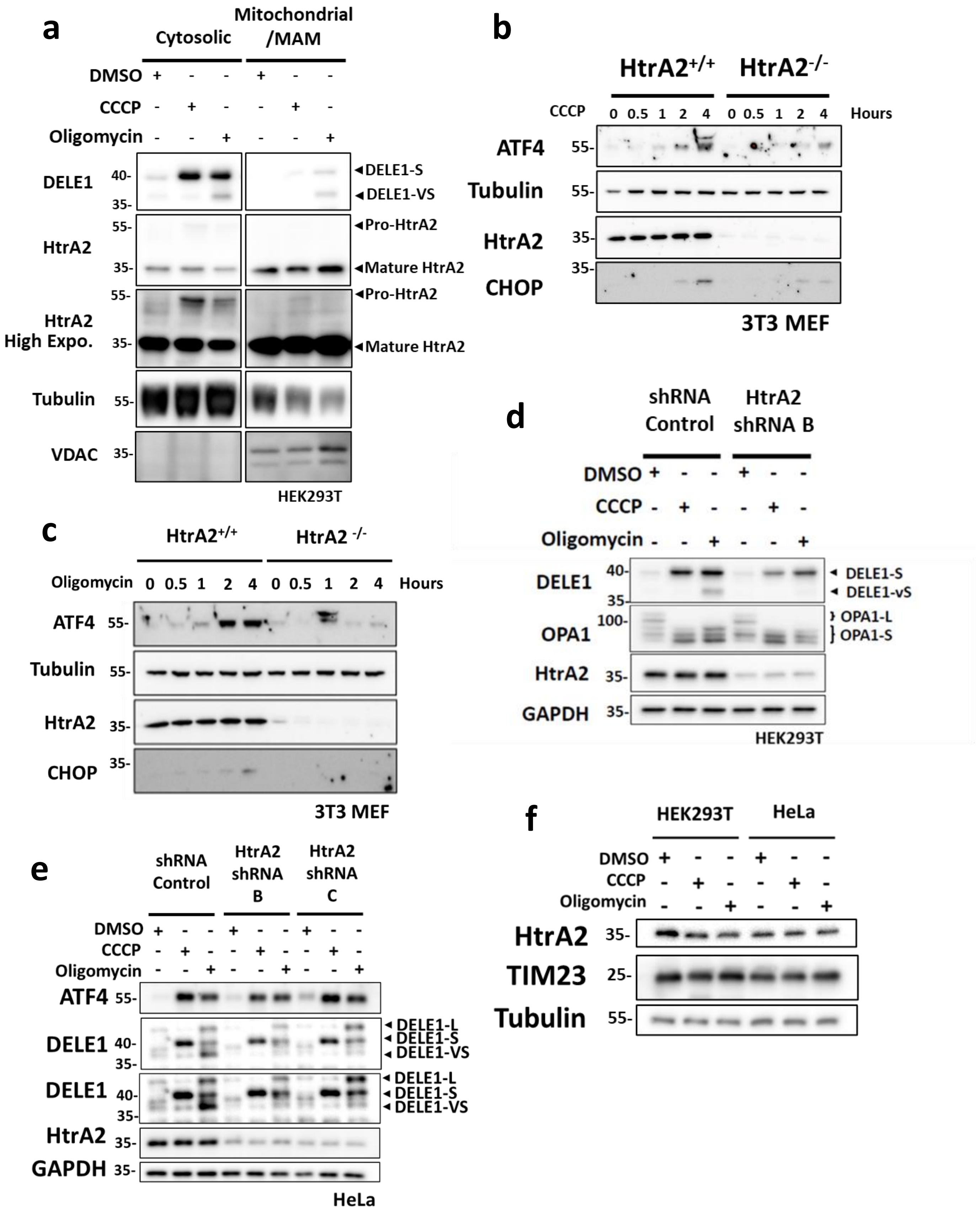

a
Cytosolic
Mitochondrial /MAM
DMSO
CCCP
Oligomycin
DELE1
DELE1-S
DELE1-VS
HtrA2
Pro-HtrA2
Mature HtrA2
HtrA2 High Expo.
Tubulin
VDAC
HEK293T
b
HtrA2+/+
HtrA2-/-
CCCP 0 0.5 1 2 4 0 0.5 1 2 4 Hours
ATF4
Tubulin
HtrA2
CHOP
3T3 MEF
c
HtrA2+/+
HtrA2 -/-
Oligomycin 0 0.5 1 2 4 0 0.5 1 2 4 Hours
ATF4
Tubulin
HtrA2
CHOP
3T3 MEF
d
shRNA Control
HtrA2 shRNA B
DMSO
CCCP
Oligomycin
DELE1
DELE1-S
DELE1-vS
OPA1
OPA1-L
OPA1-S
HtrA2
GAPDH
HEK293T
e
shRNA Control
HtrA2 shRNA B
HtrA2 shRNA C
DMSO
CCCP
Oligomycin
ATF4
DELE1
DELE1-L
DELE1-S
DELE1-VS
HtrA2
GAPDH
HeLa
f
HEK293T
HeLa
DMSO
CCCP
Oligomycin
HtrA2
TIM23
Tubulin

Supplementary Fig 5. HTRA2 plays a critical role in DELE1-VS generation.

a Mitochondrial fractionation of WT HEK293T cells lysates following 4 hours of DMSO, CCCP (20 μM) or oligomycin (10 μM) and analyzed by WB. **b** WT ($HtrA2^{+/+}$) and $HtrA2^{-/-}$ 3T3 MEF cells were treated with 20 μM CCCP for the indicated time and whole-cell lysates were analyzed by WB. **c** WT ($HtrA2^{+/+}$) and $HtrA2^{-/-}$ 3T3 MEF cells were treated with 10 μM oligomycin for the indicated time and whole-cell lysates were analyzed by WB. **d** SC and shHtrA2 B HEK293T cells were treated with DMSO, CCCP (20 μM) or oligomycin (10 μM) for 4 hours. Whole-cell lysates were analyzed by WB. **e** SC, shHtrA2 B and C HeLa cells were treated with DMSO, CCCP (20 μM) or oligomycin (10 μM) for 4 hours. Whole-cell lysates were analyzed by WB. **f** WT HEK293T and HeLa cells were treated with DMSO, CCCP (20 μM) and oligomycin (10 μM) to 4 hours and whole-cell lysates were analyzed by WB.

Original Blots

Fig. 1a

ATF4

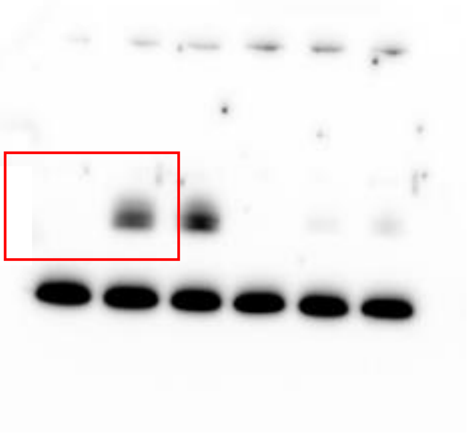

DELE1

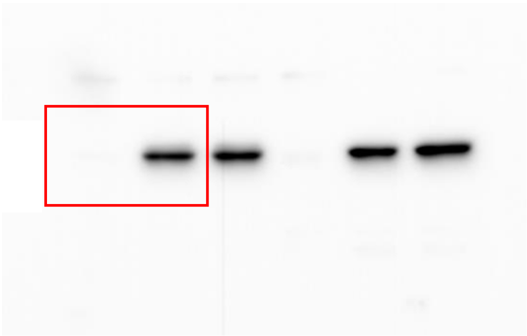

GAPDH

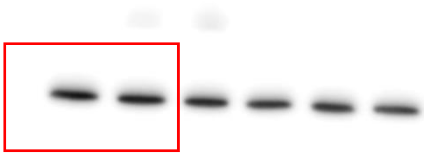

Fig. 1b

DELE1

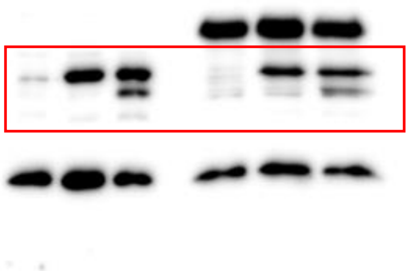

GAPDH

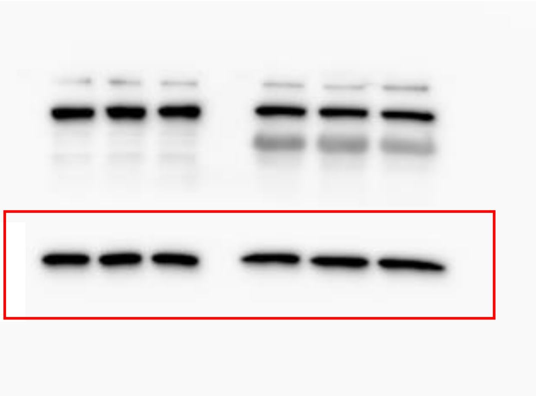

Fig. 1c

ATF4

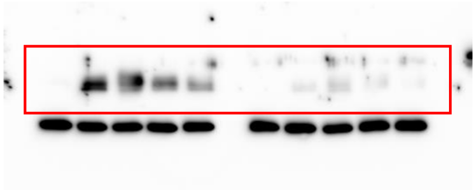

GAPDH

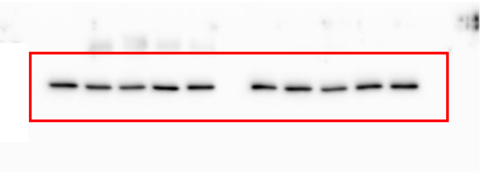

DELE1

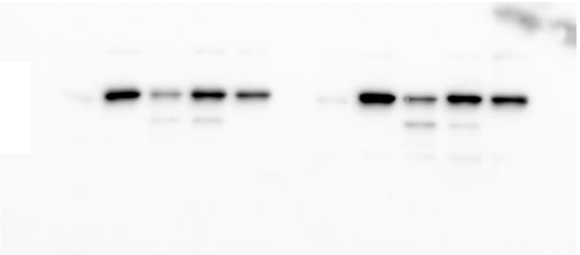

Fig. 1d

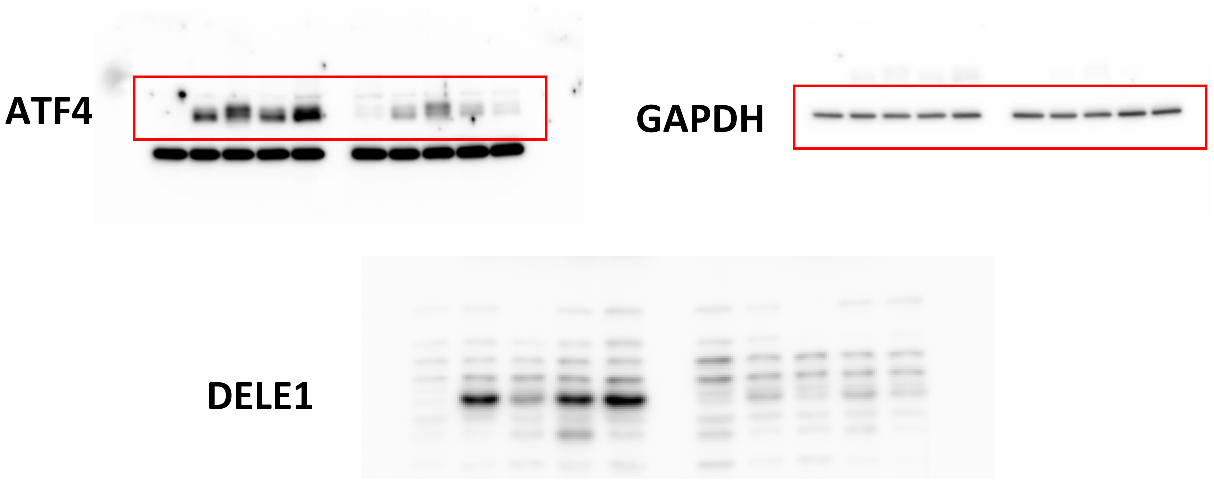

Fig. 1e

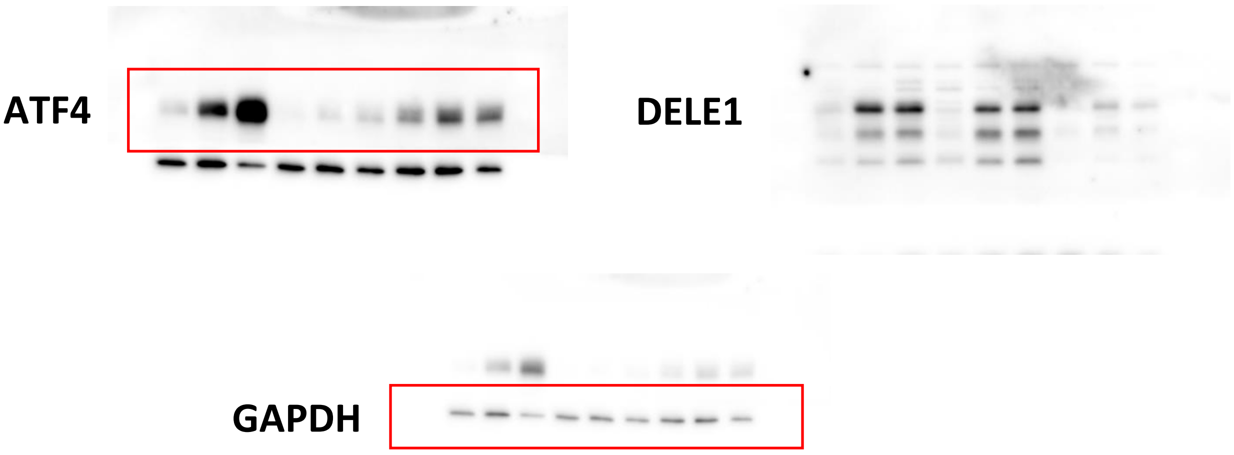

Fig. 1f

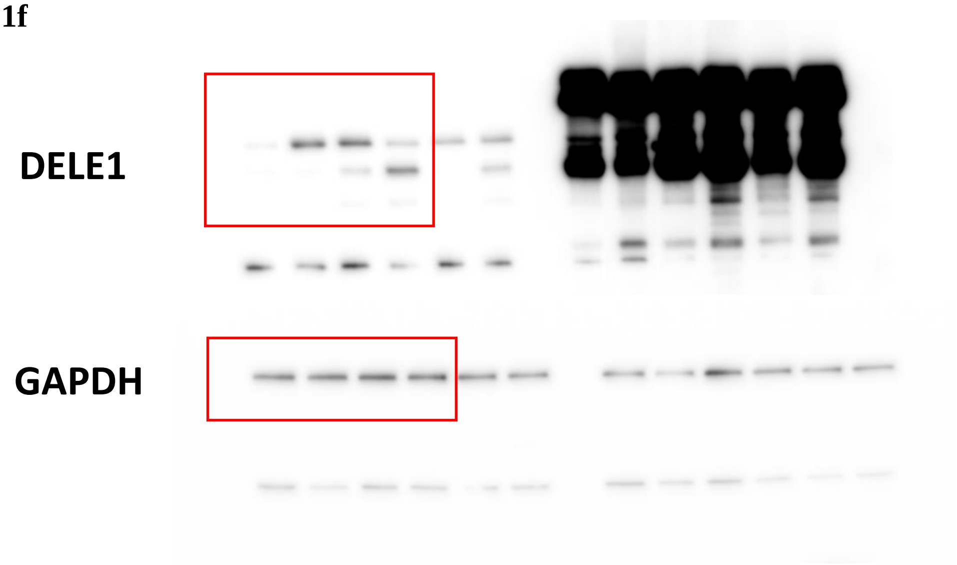

Fig. 1g

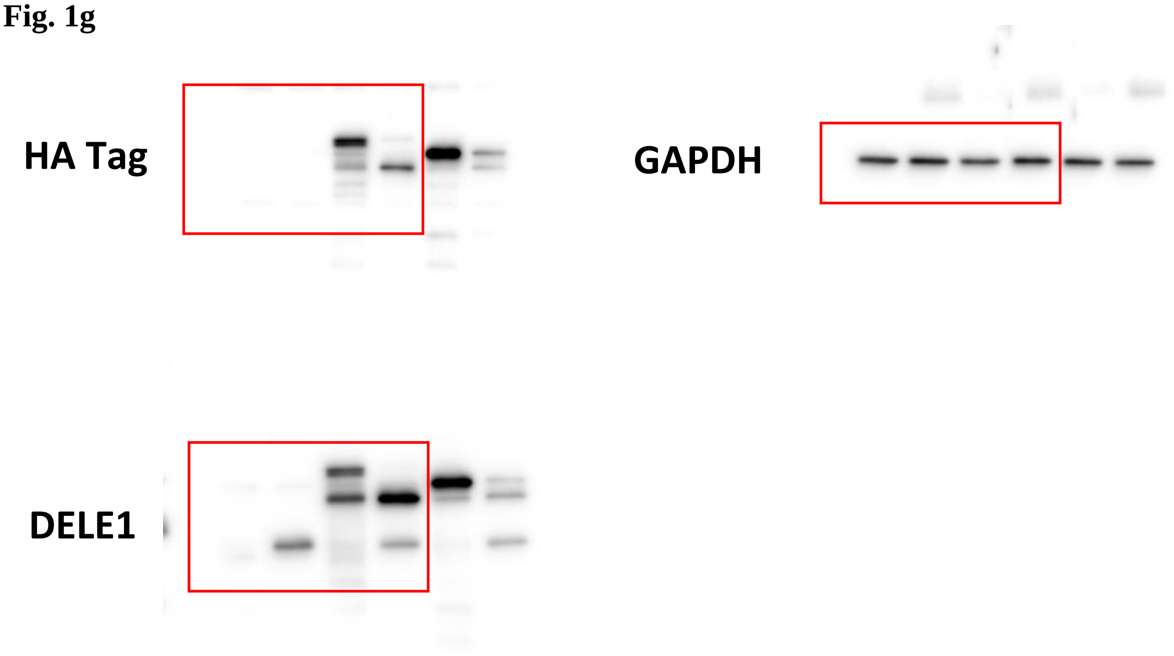

Fig. 1h

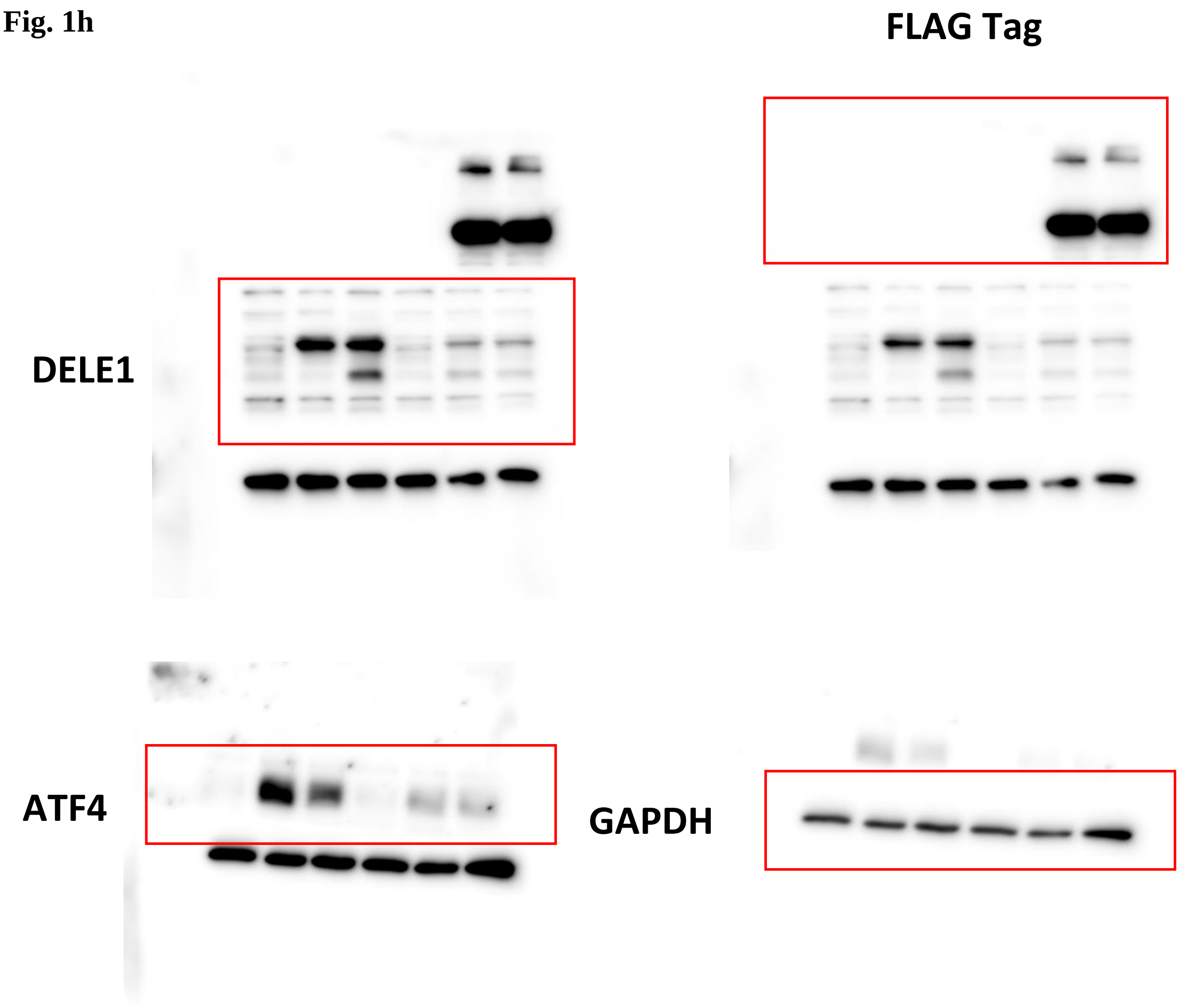

Fig. 2a

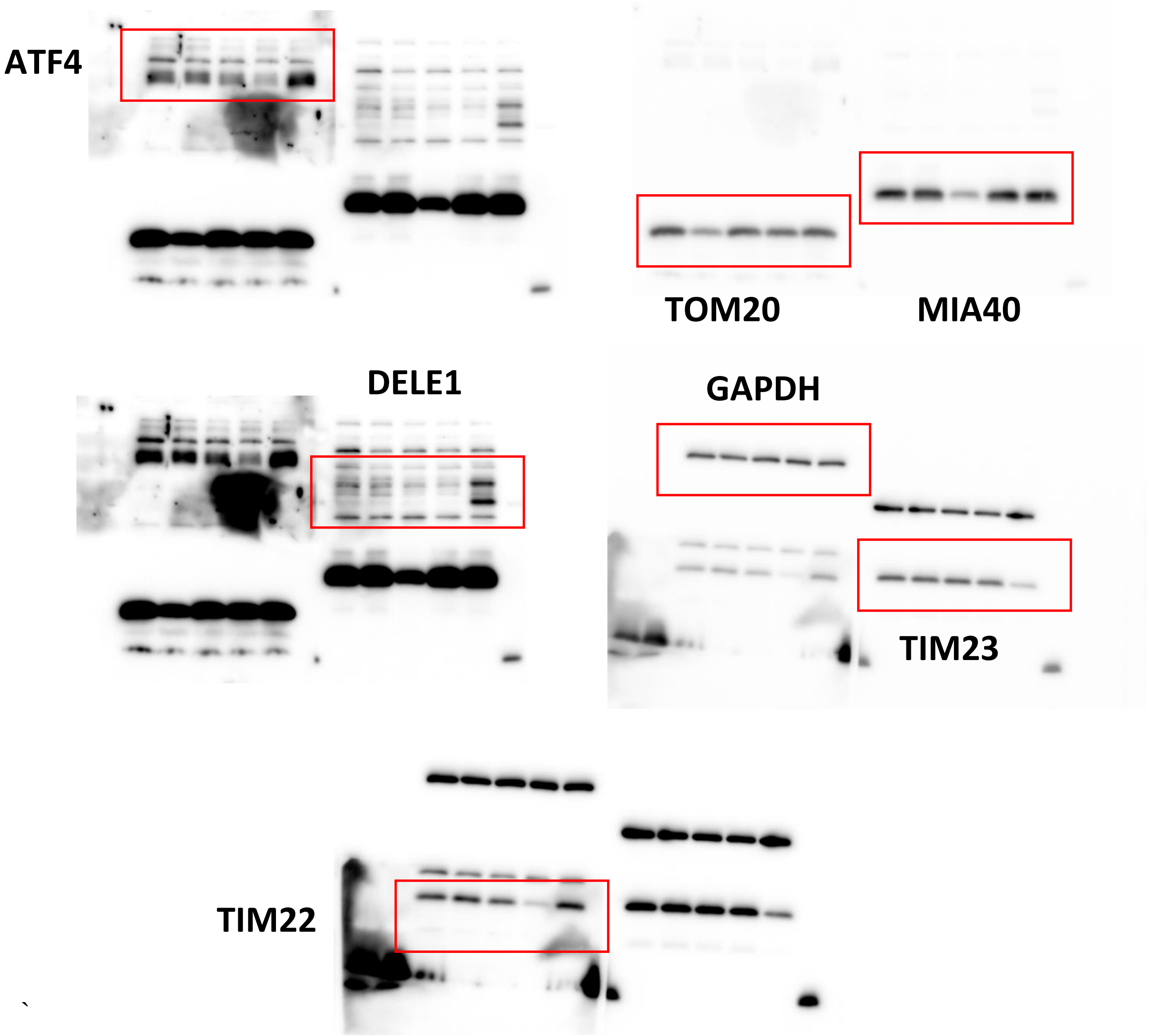

Fig. 2b

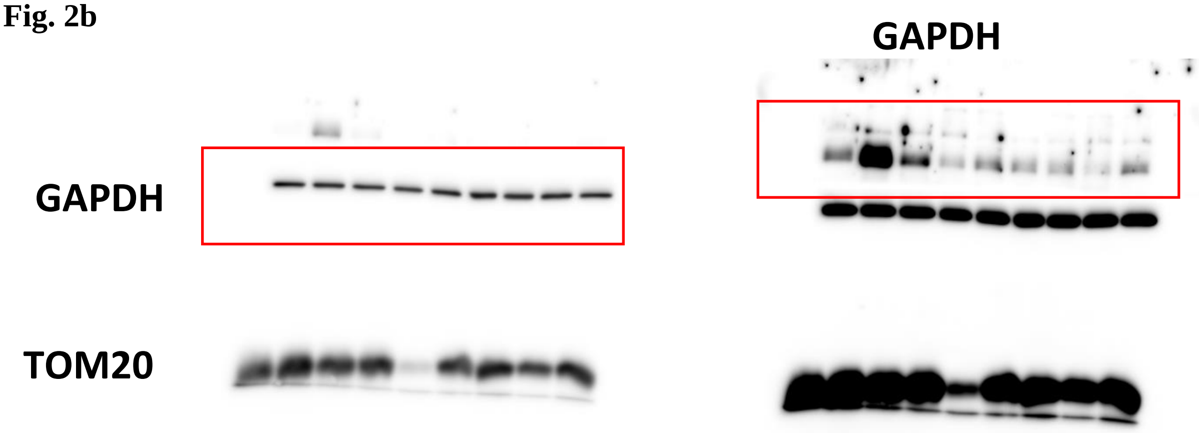

Fig. 2b

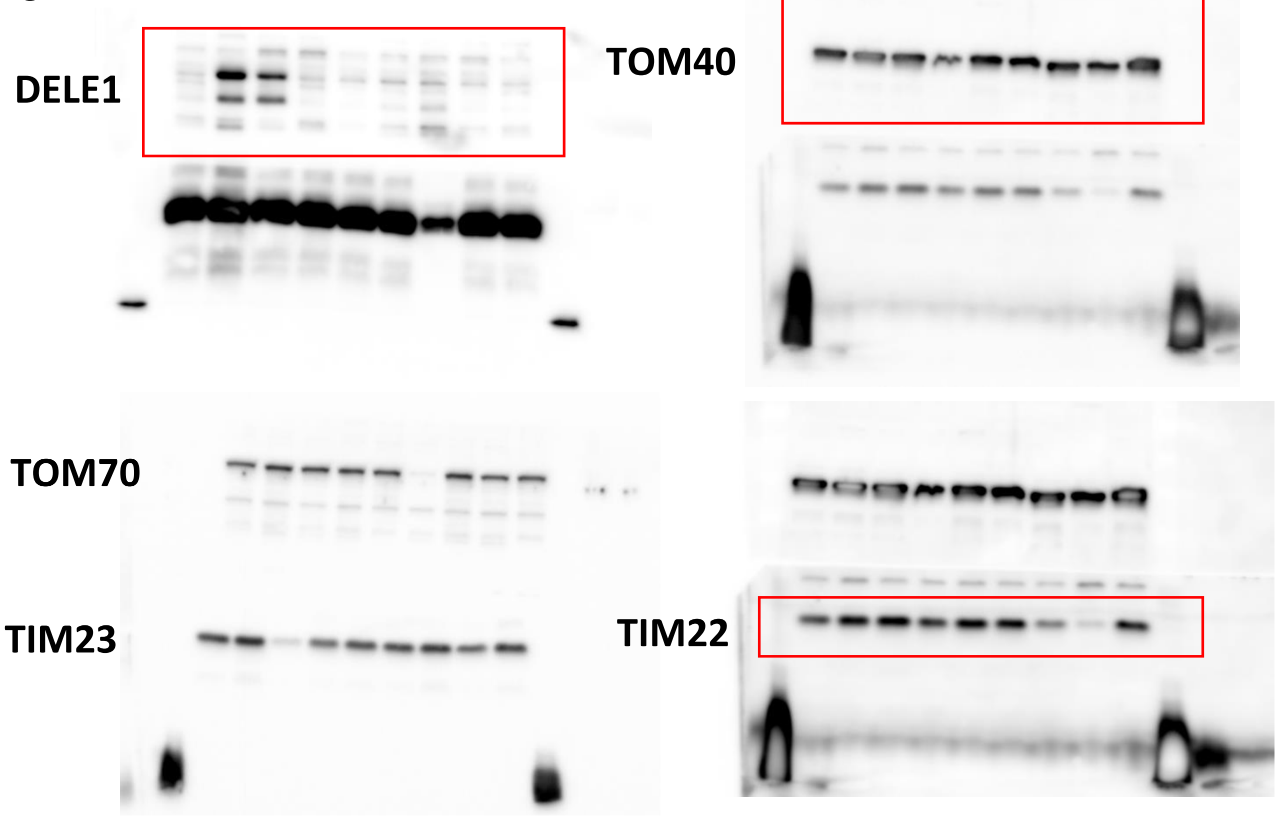

Fig. 2c

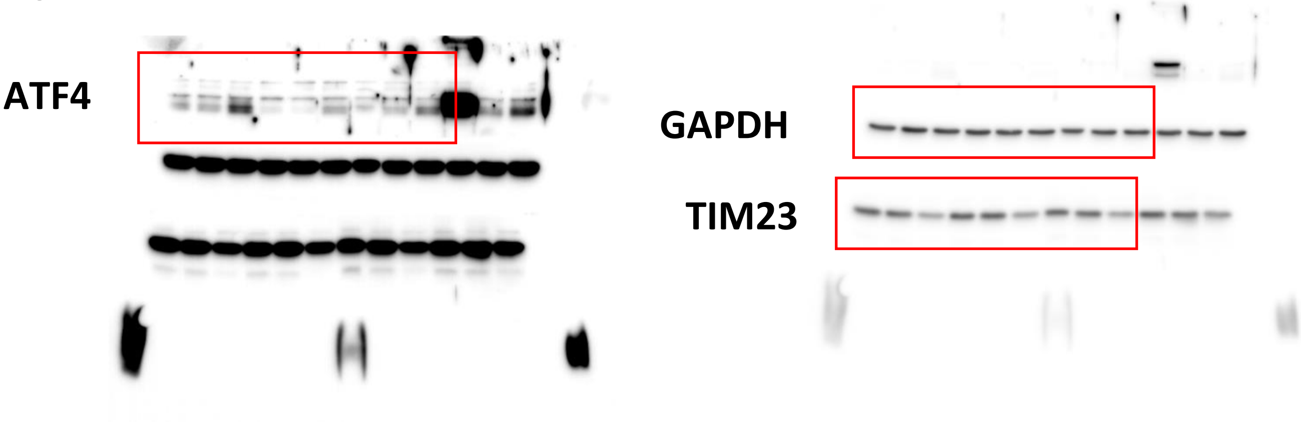

Fig. 3c

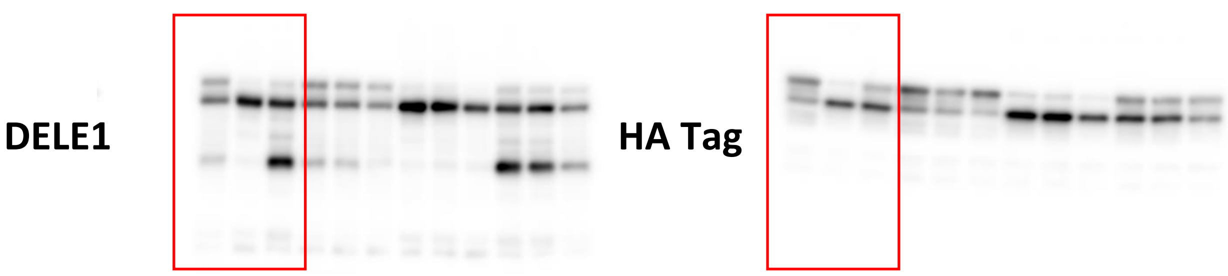

GAPDH

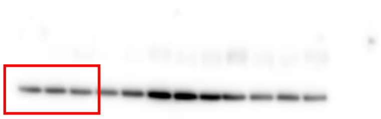

Fig. 3d

GAPDH

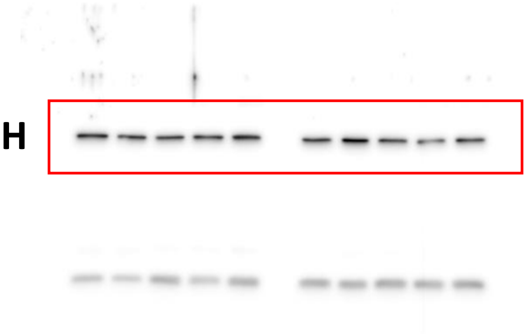

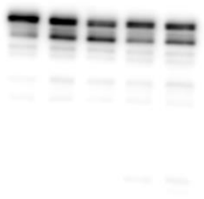

HA Tag

Fig. 3e

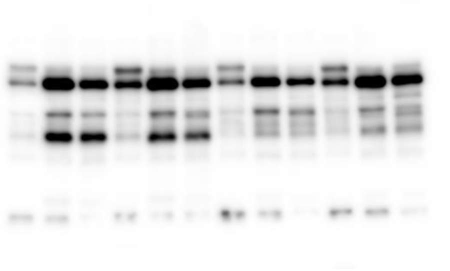

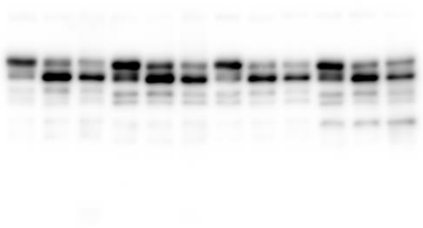

DELE1

HA Tag

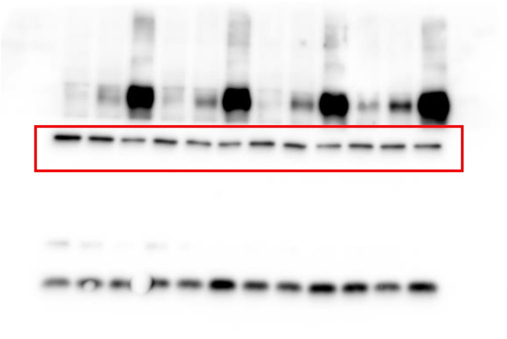

GAPDH

Fig. 3h

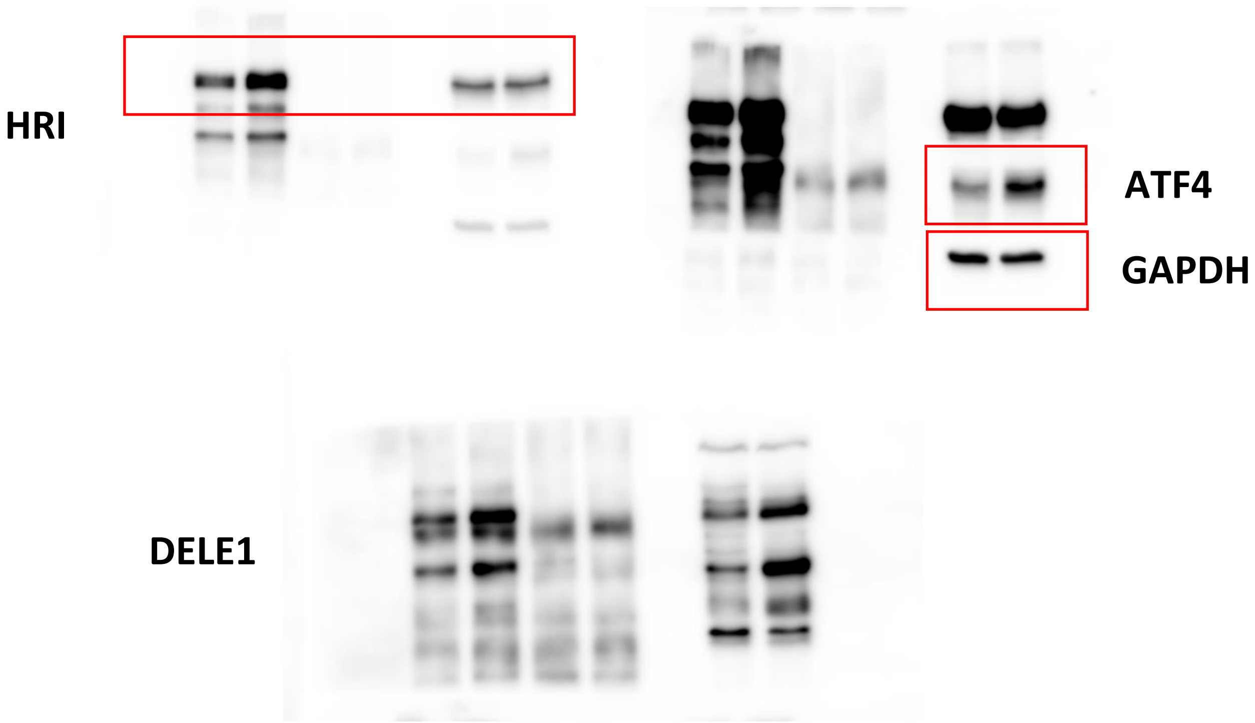

Fig. 4a

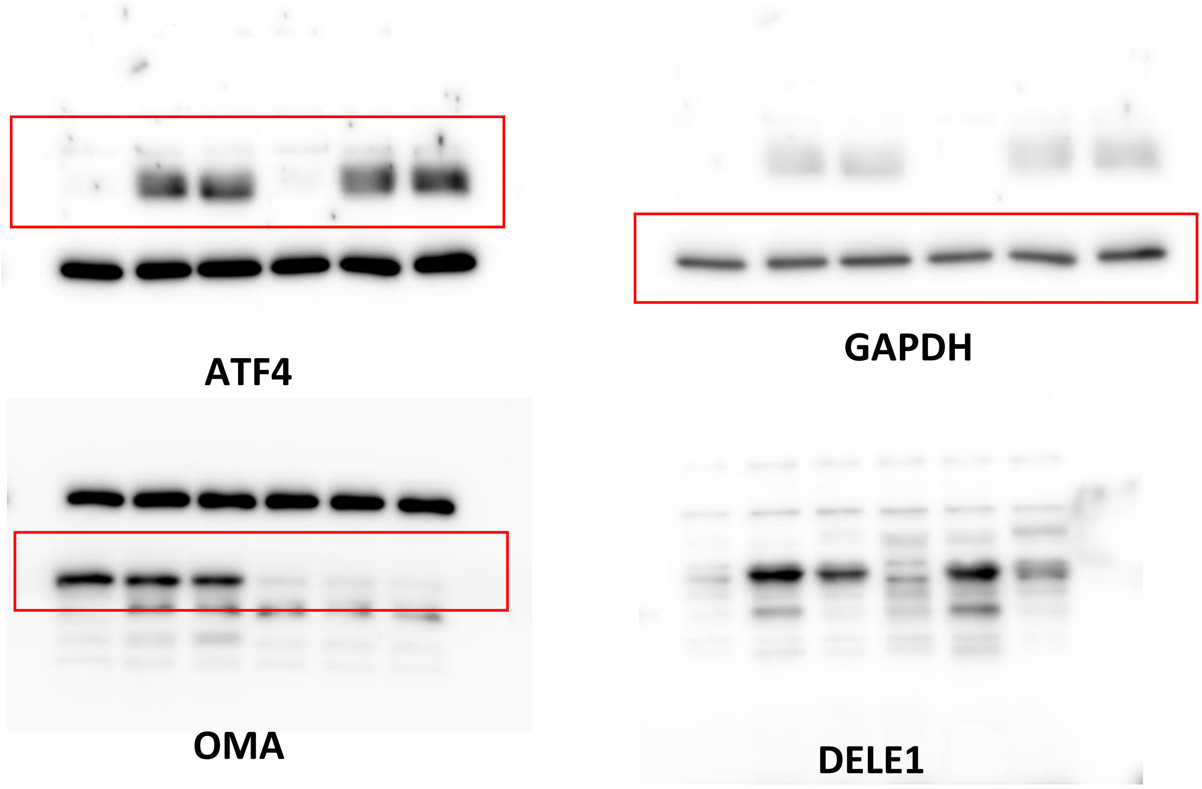

Fig. 4b

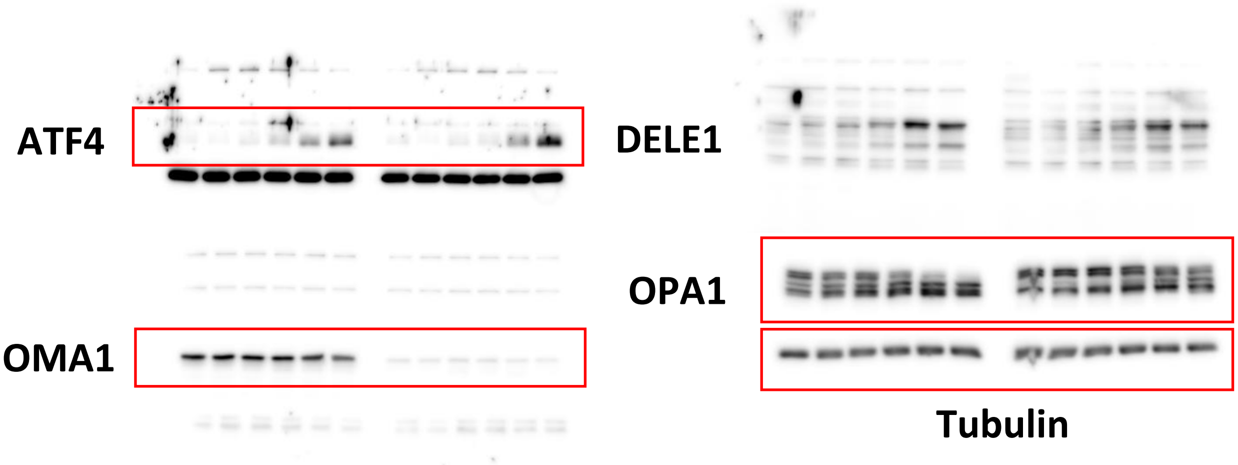

Fig. 4c

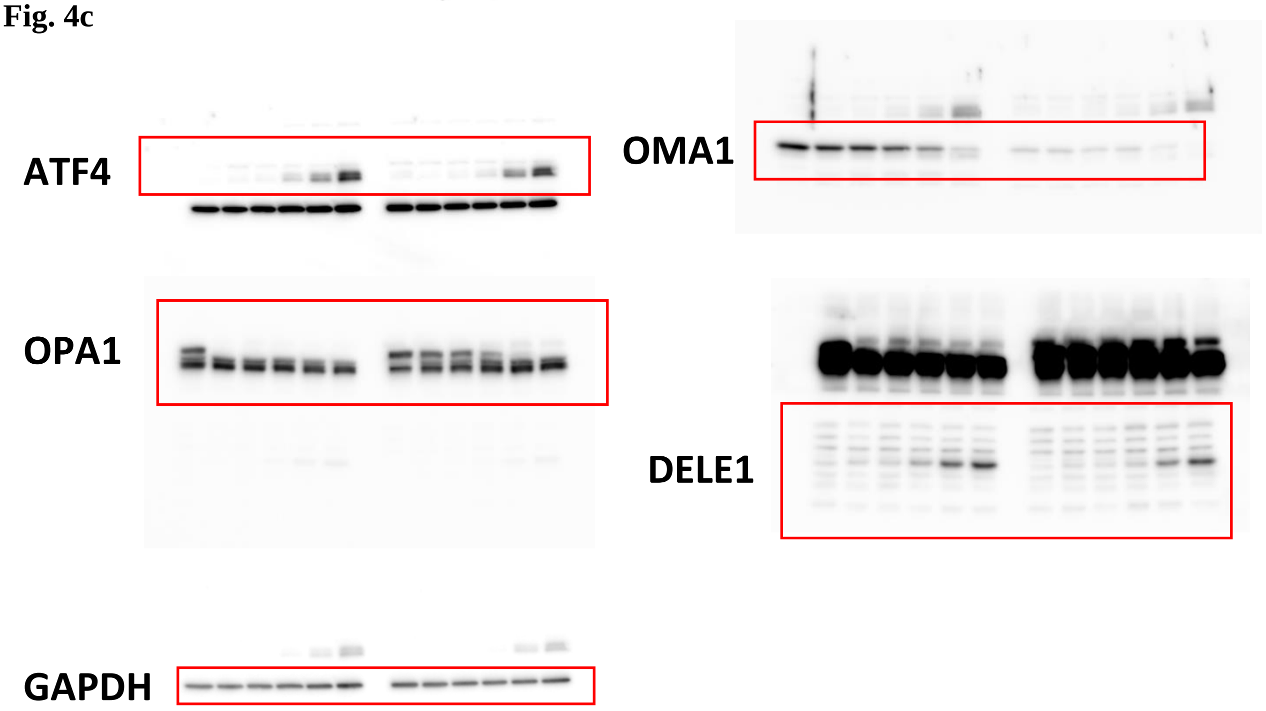

Fig. 4d

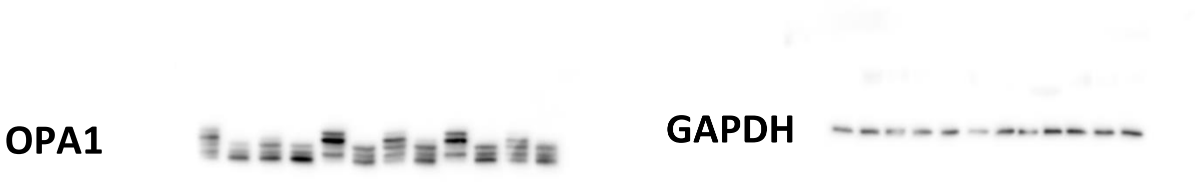

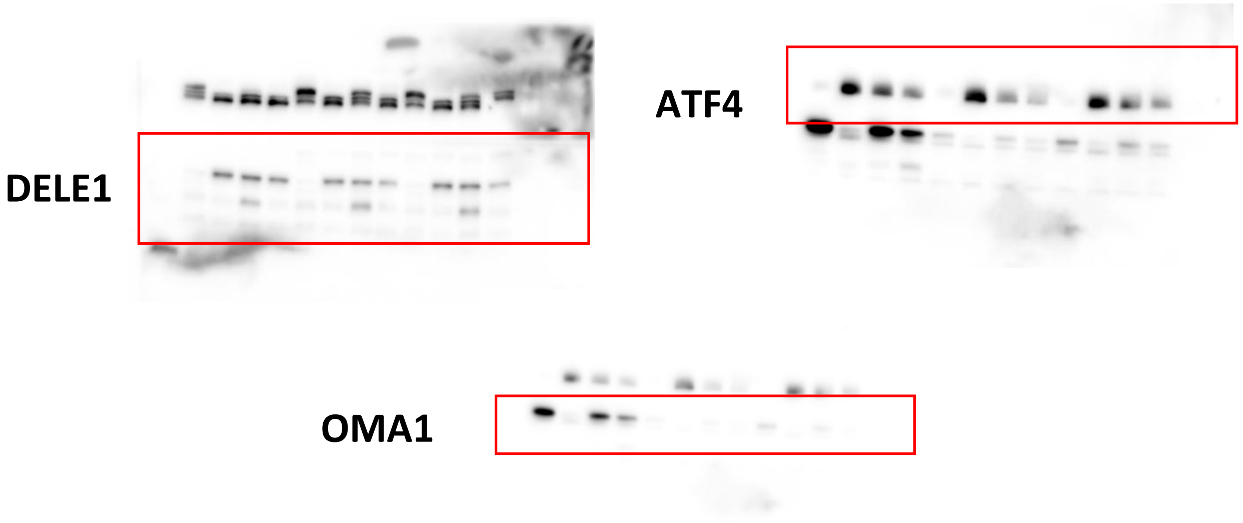

Fig. 4e

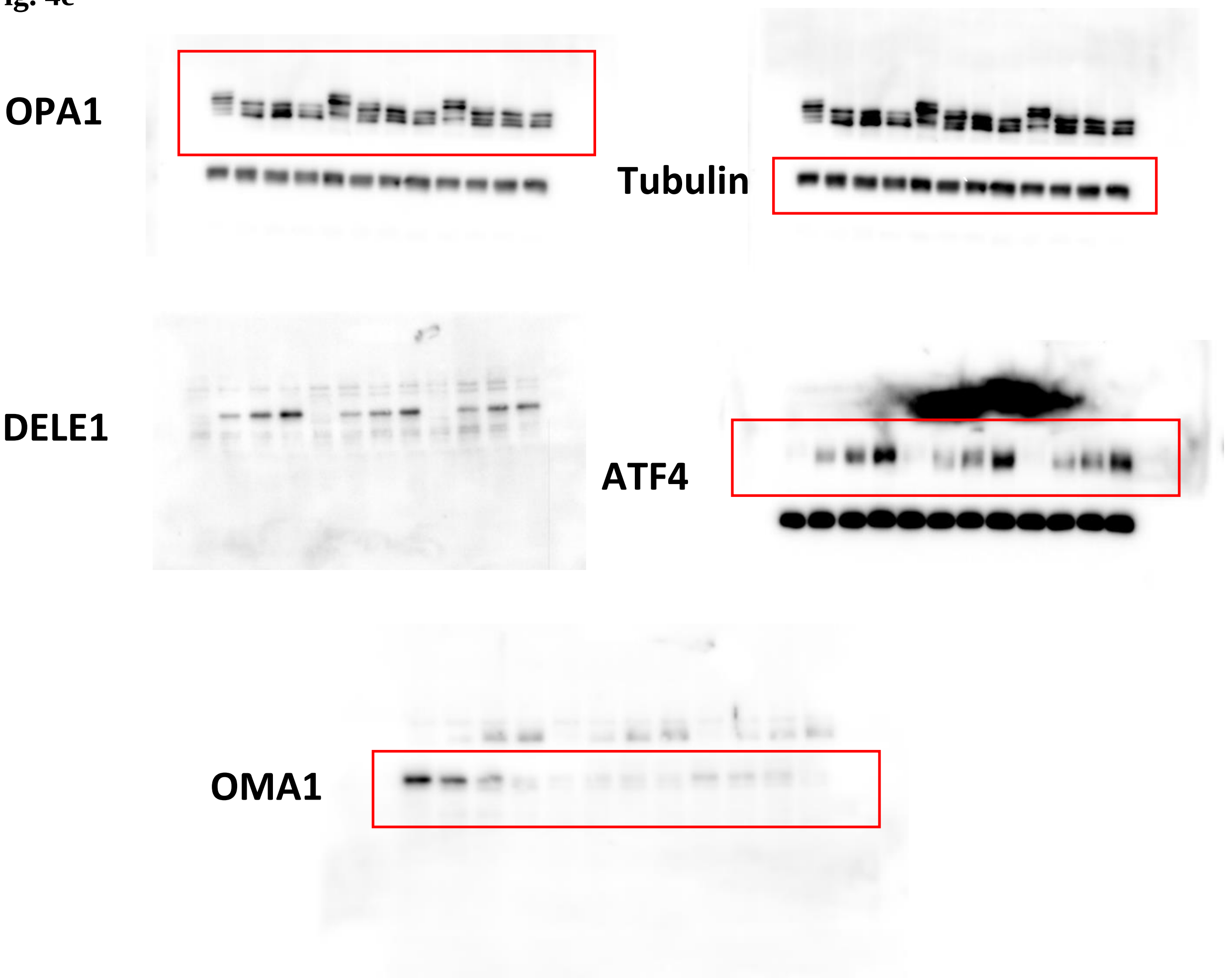

Fig. 4f

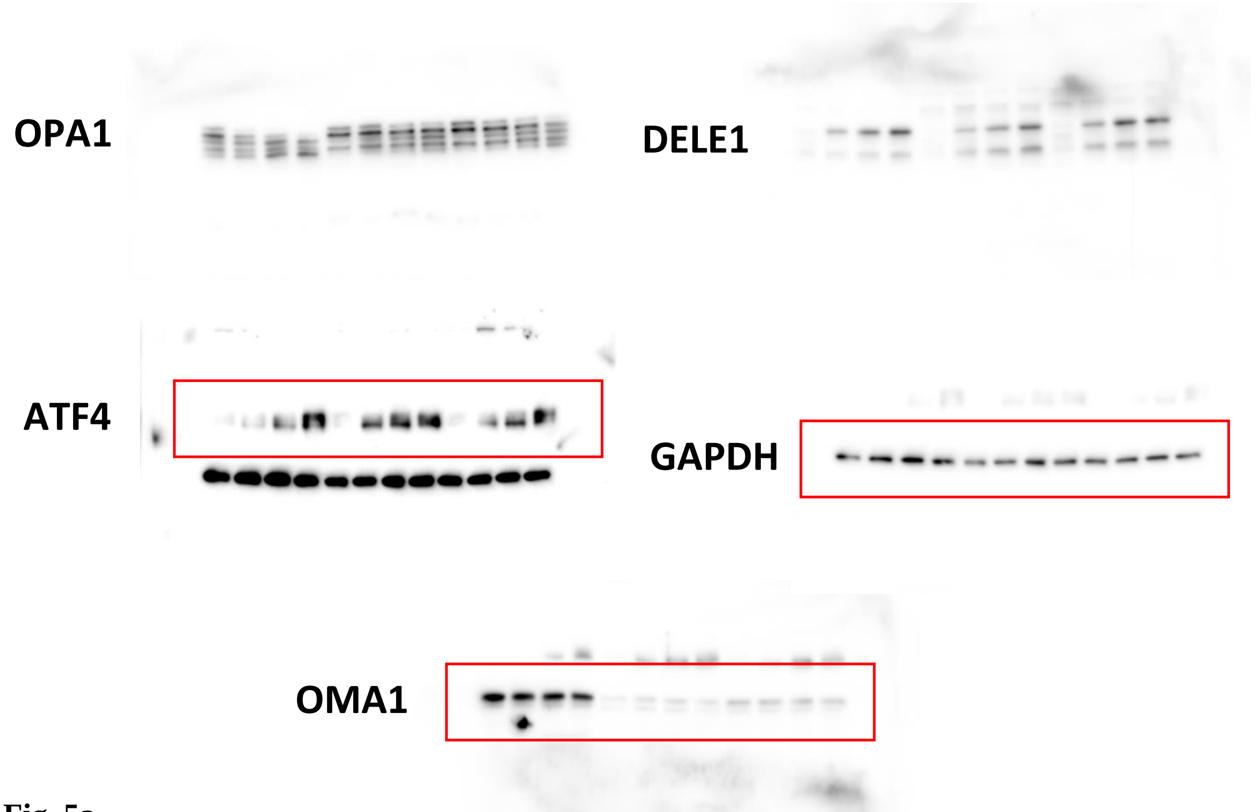

Fig. 5a

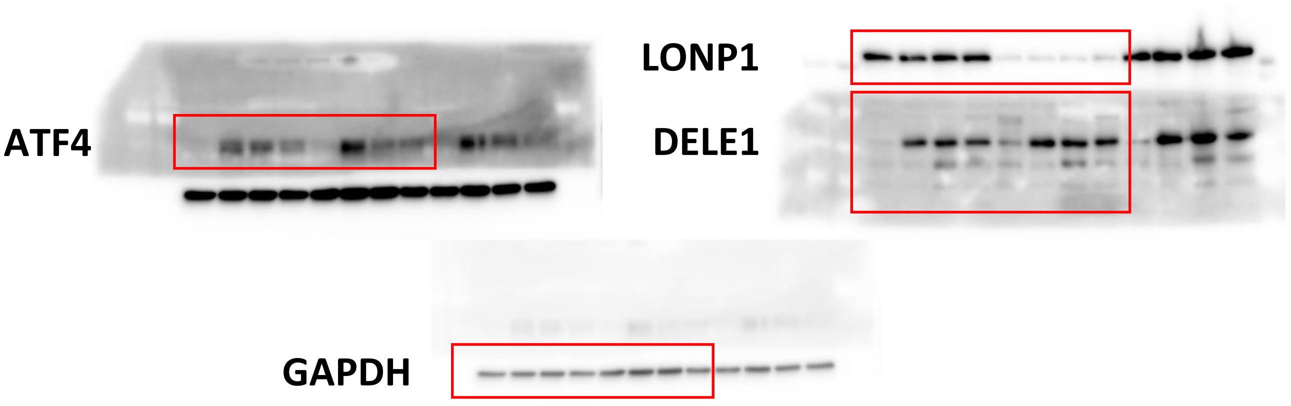

Fig. 5b

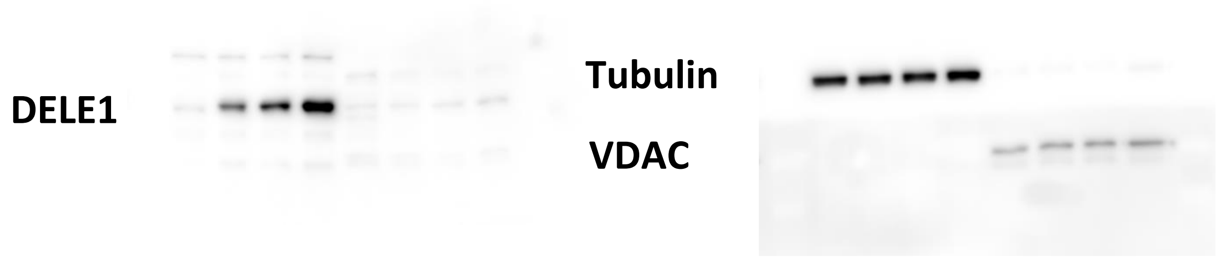

Fig. 5c

DELE1

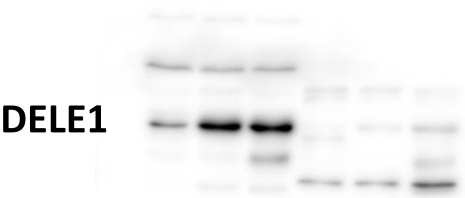

Tubulin

VDAC

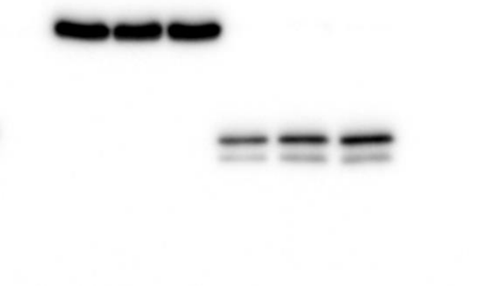

Fig. 5d

DELE1

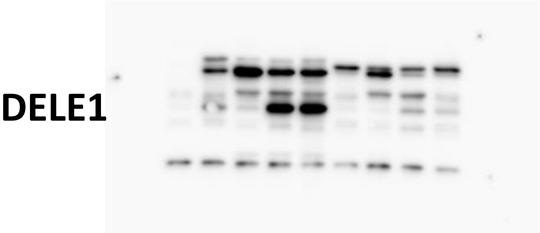

GAPDH

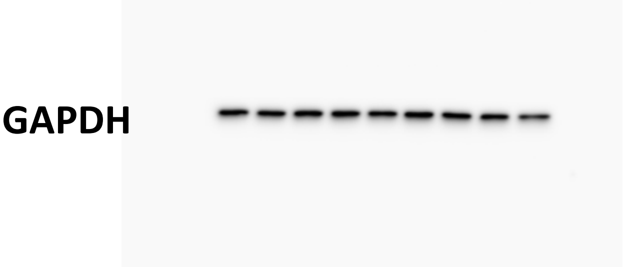

Fig. 6a

GAPDH

DELE1

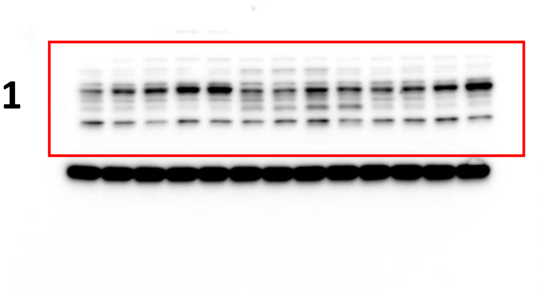

Fig. 6b

DELE1

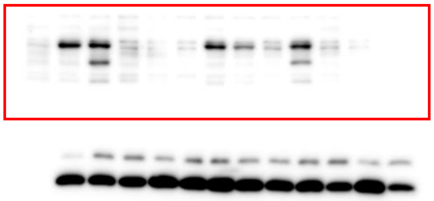

GAPDH

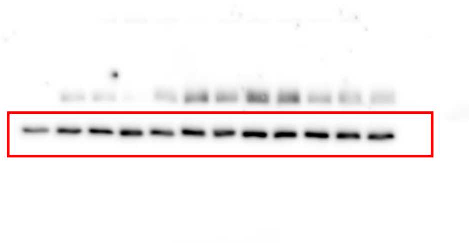

OPA1

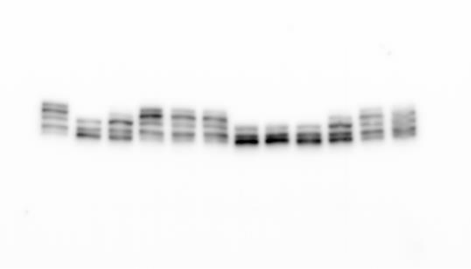

Fig. 6c

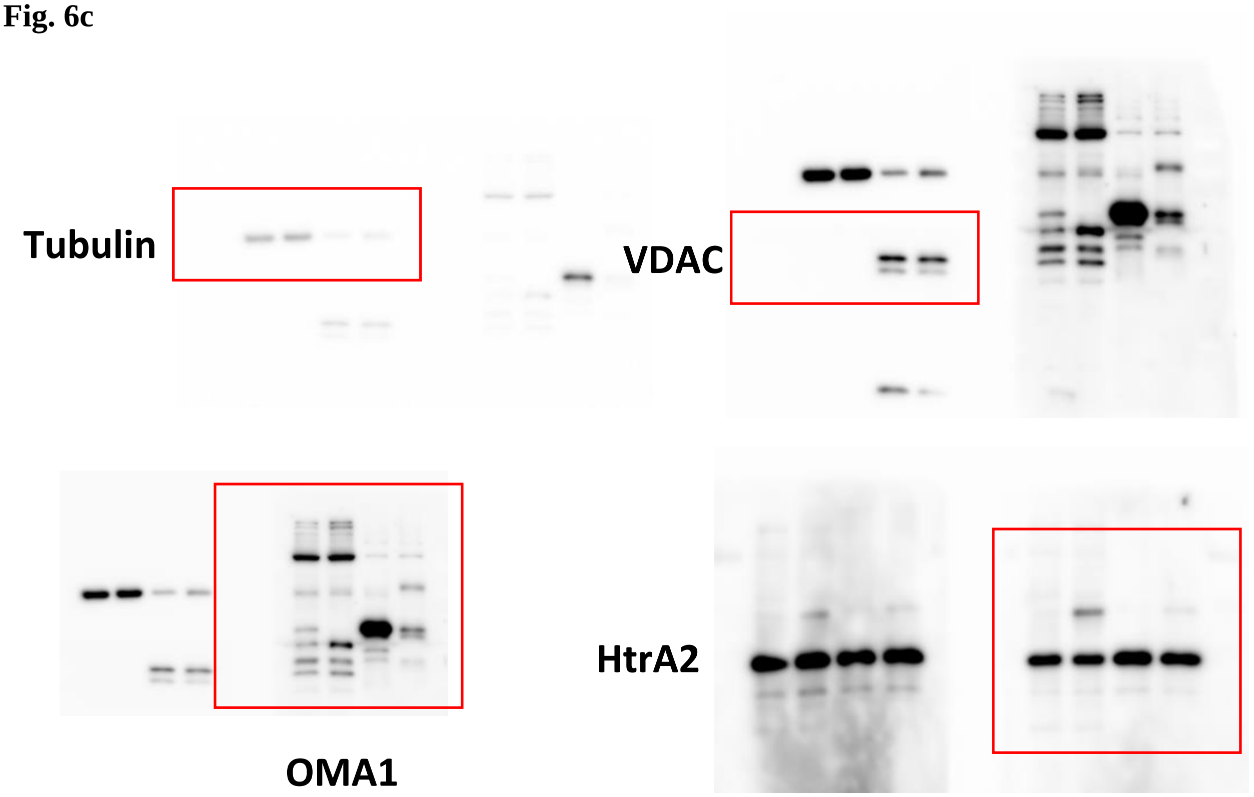

Fig. 6d

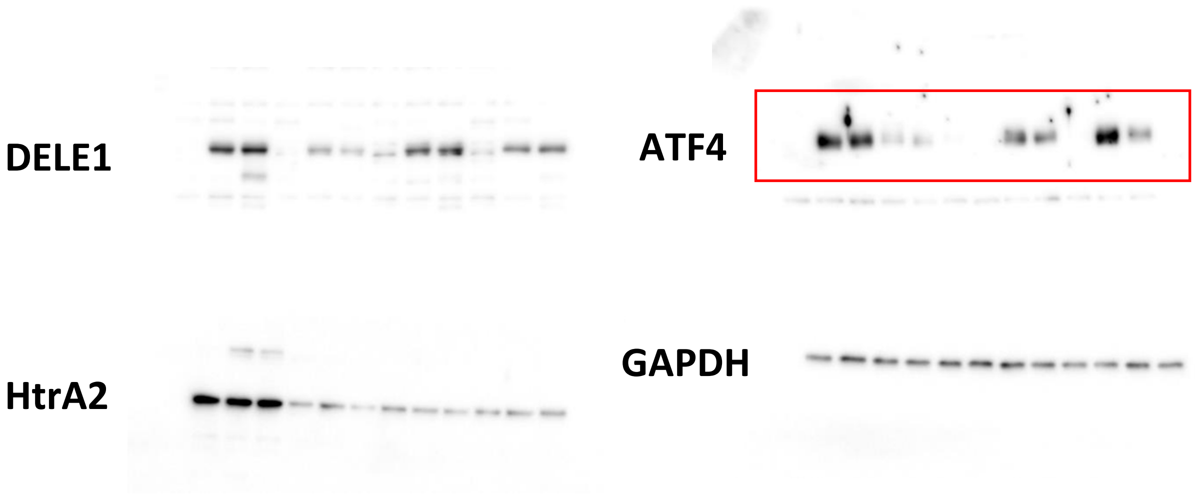

Fig. 6e

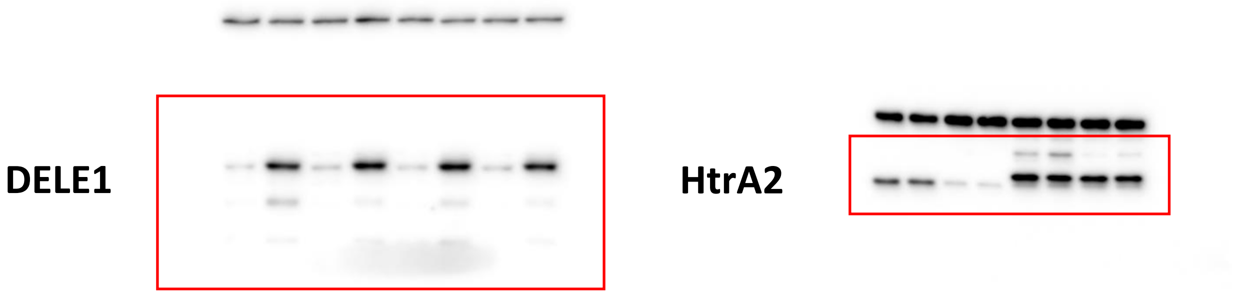

Tubulin

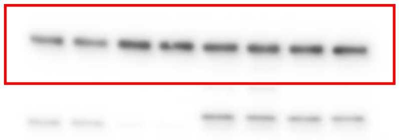

Fig. 6f

DELE1

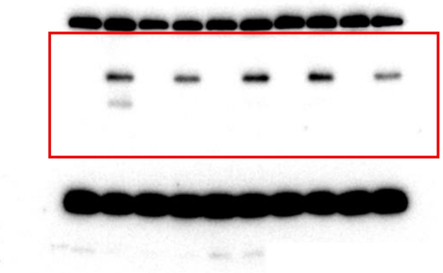

HtrA2

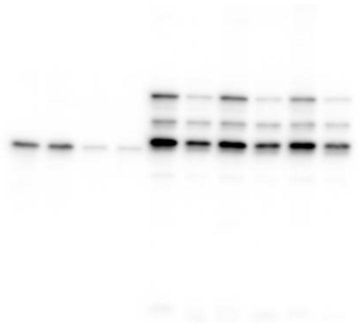

GAPDH

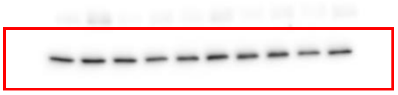

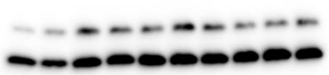

Supplementary Fig. 1a

DELE1

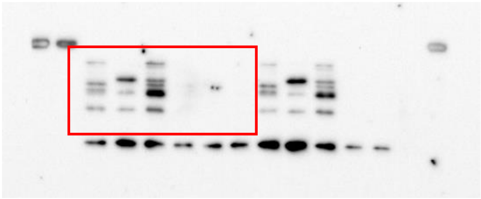

GAPDH

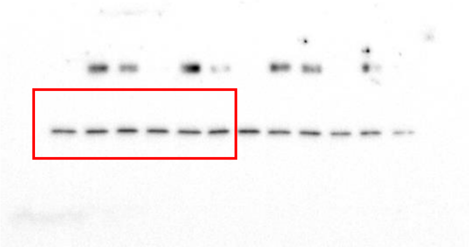

Supplementary Fig. 1b

DELE1

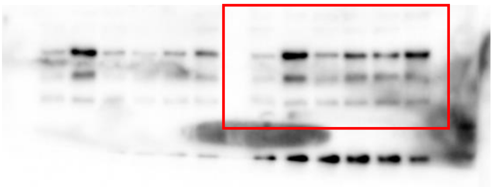

GAPDH

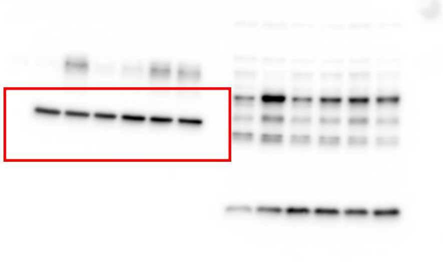

Supplementary Fig. 1c

ATF4

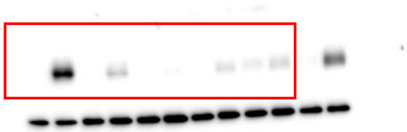

GAPDH

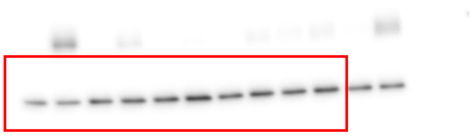

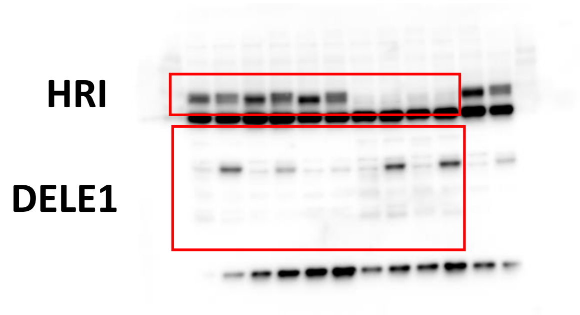

Supplementary Fig. 1d

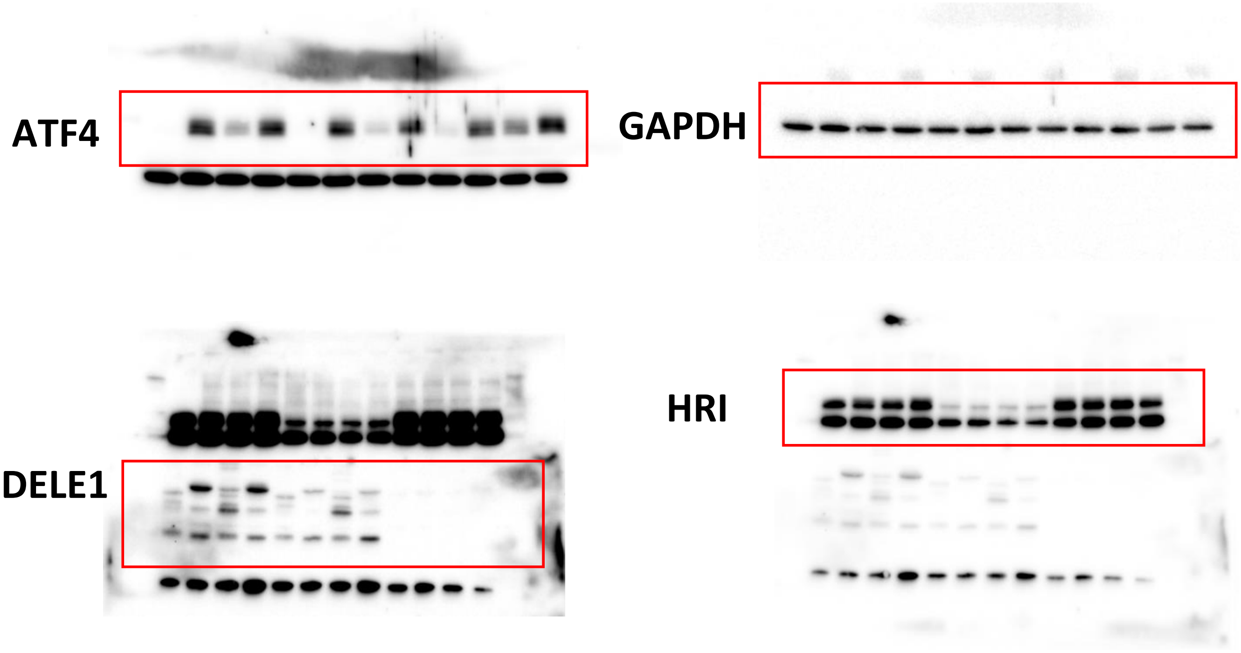

Supplementary Fig. 1e

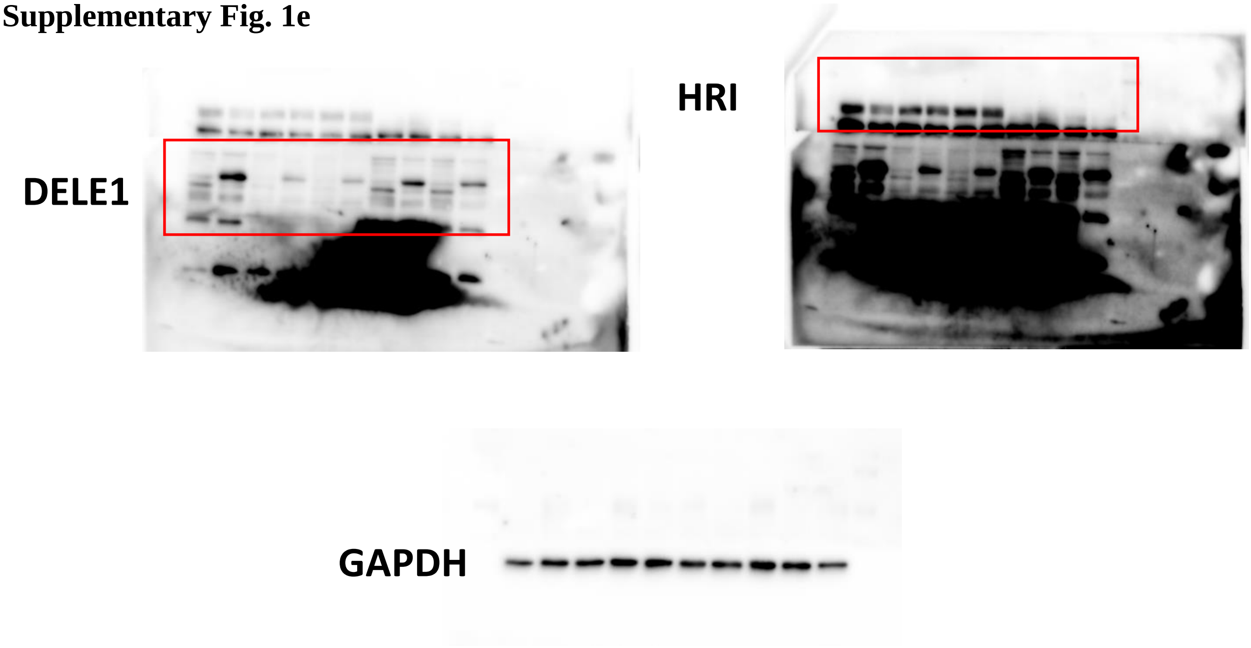

Supplementary Fig. 1f

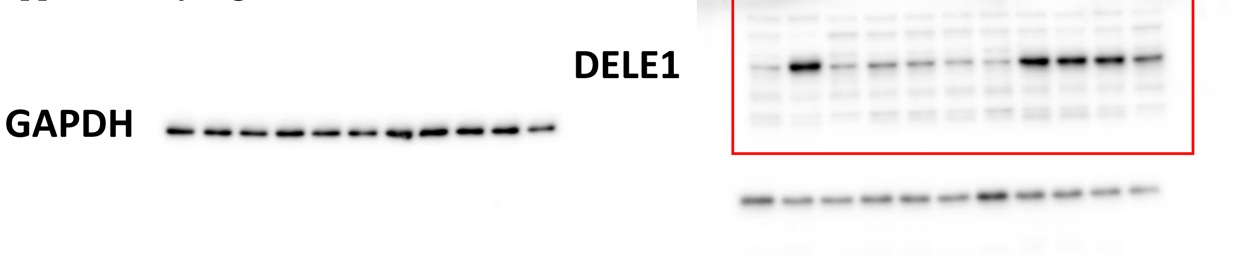

Supplementary Fig. 1g

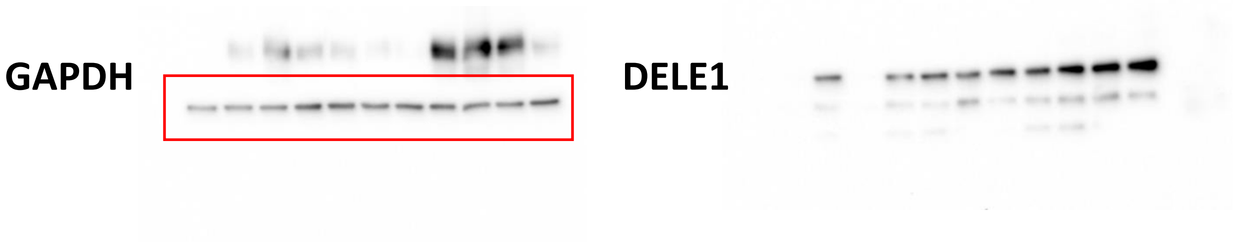

Supplementary Fig. 1h

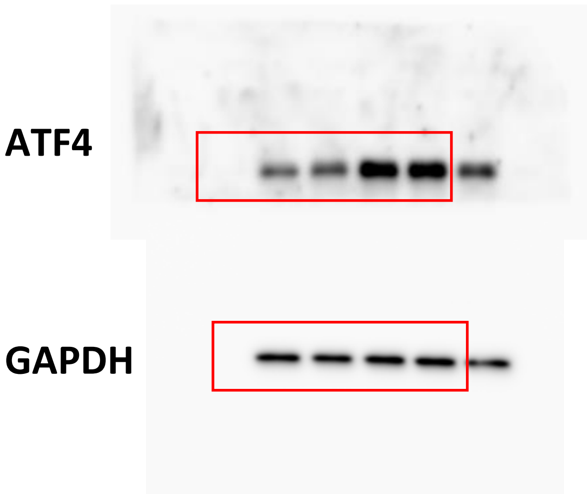

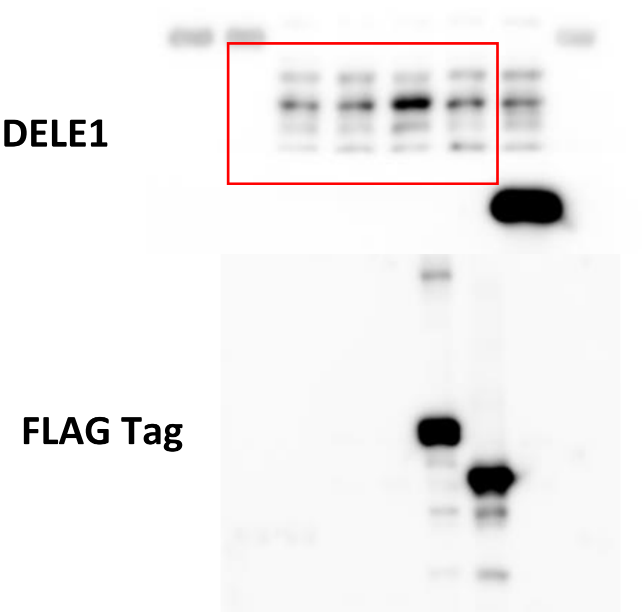

Supplementary Fig. 2

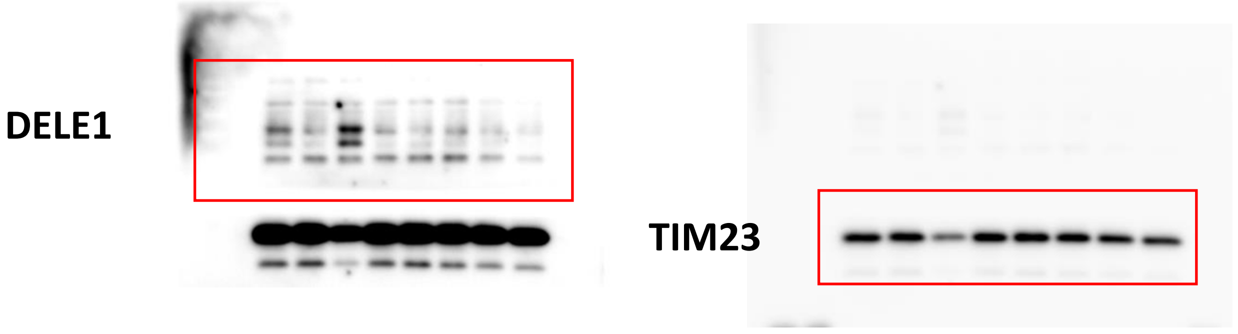

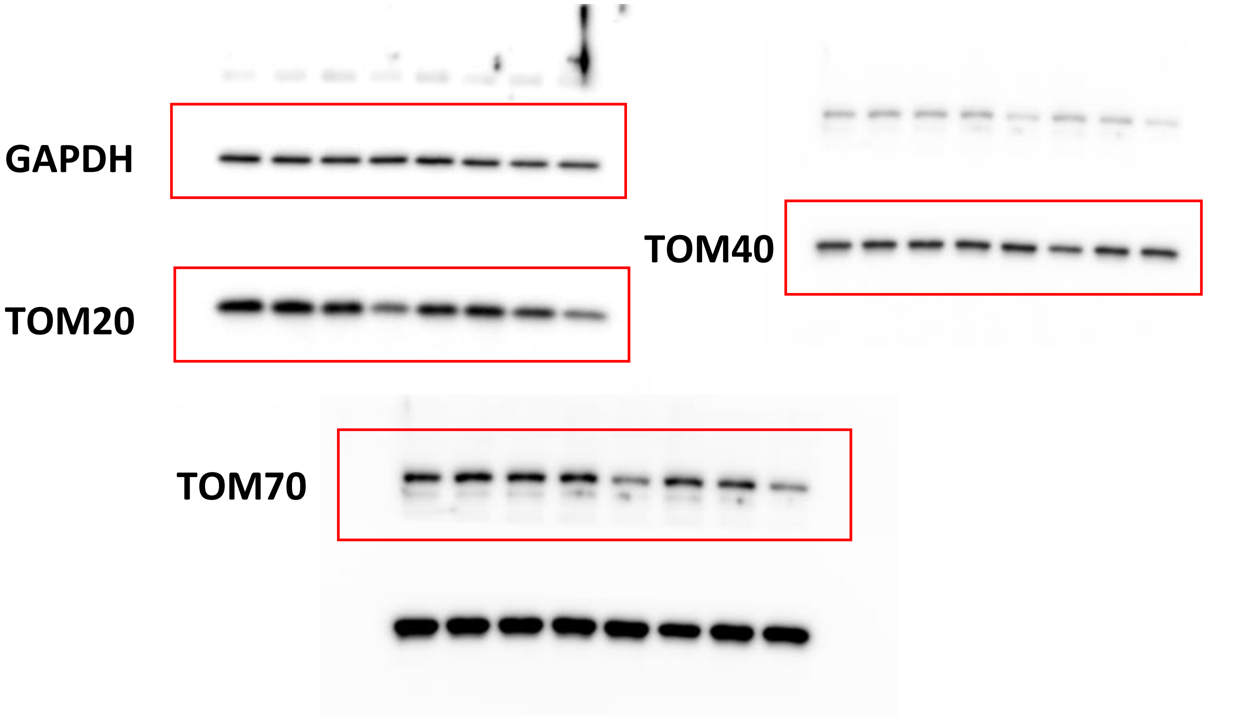

Supplementary Fig. 3a

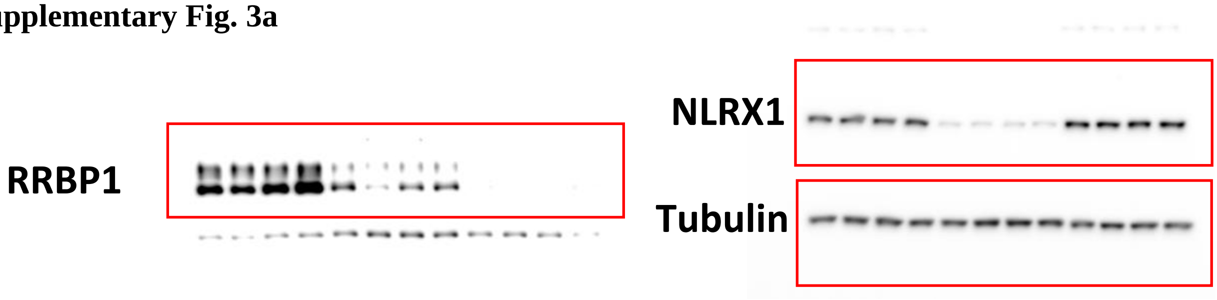

Supplementary Fig. 3b

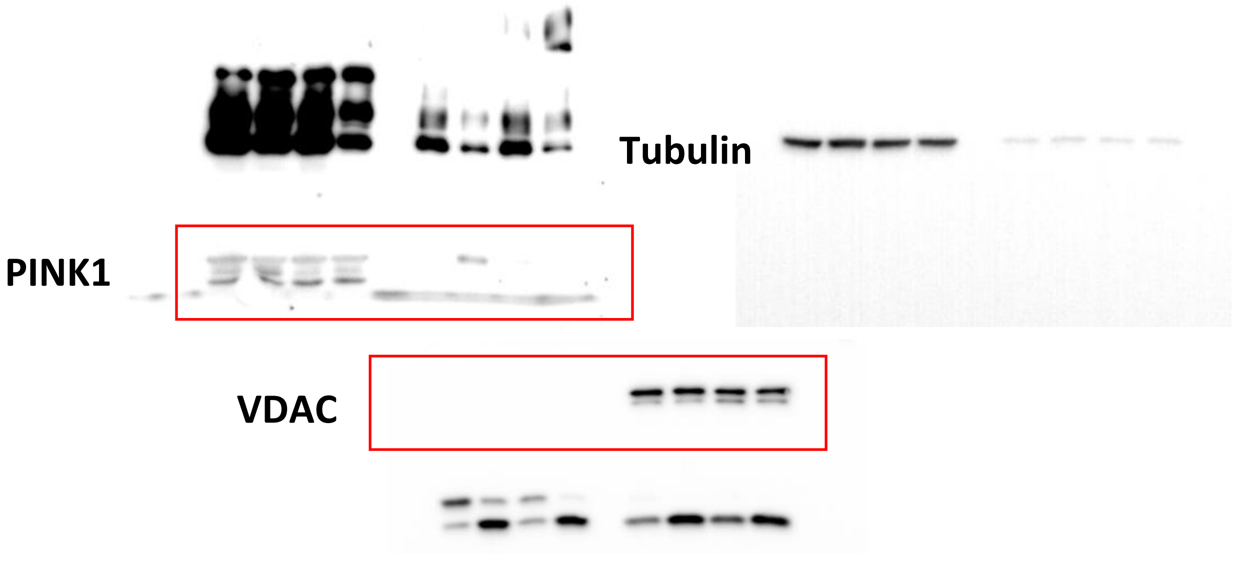

Supplementary Fig. 3c

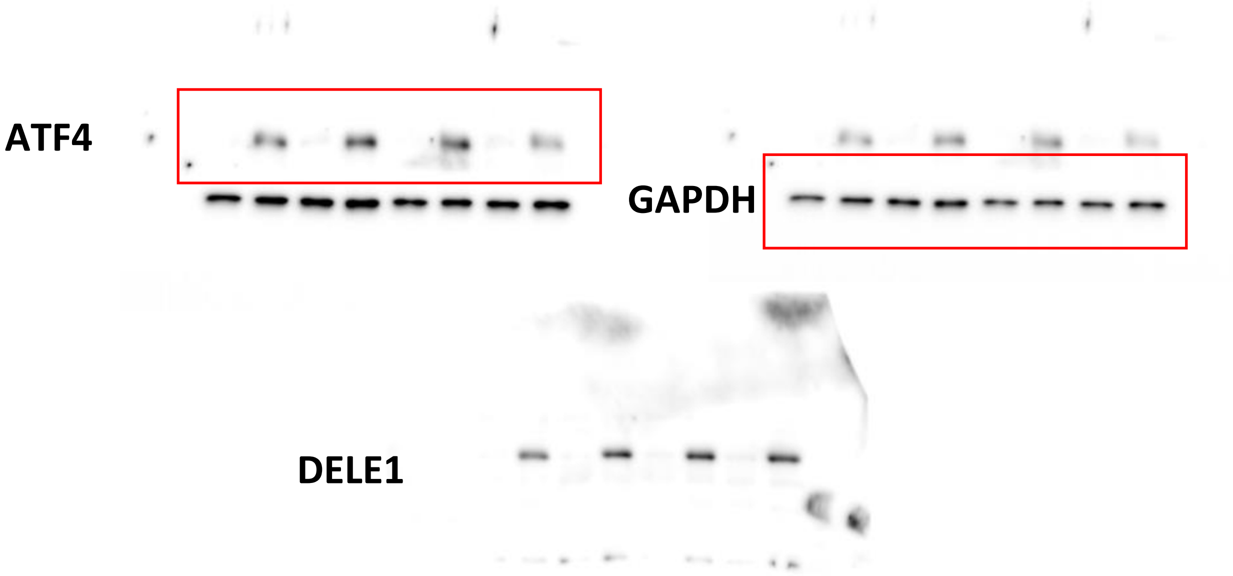

Supplementary Fig. 3d

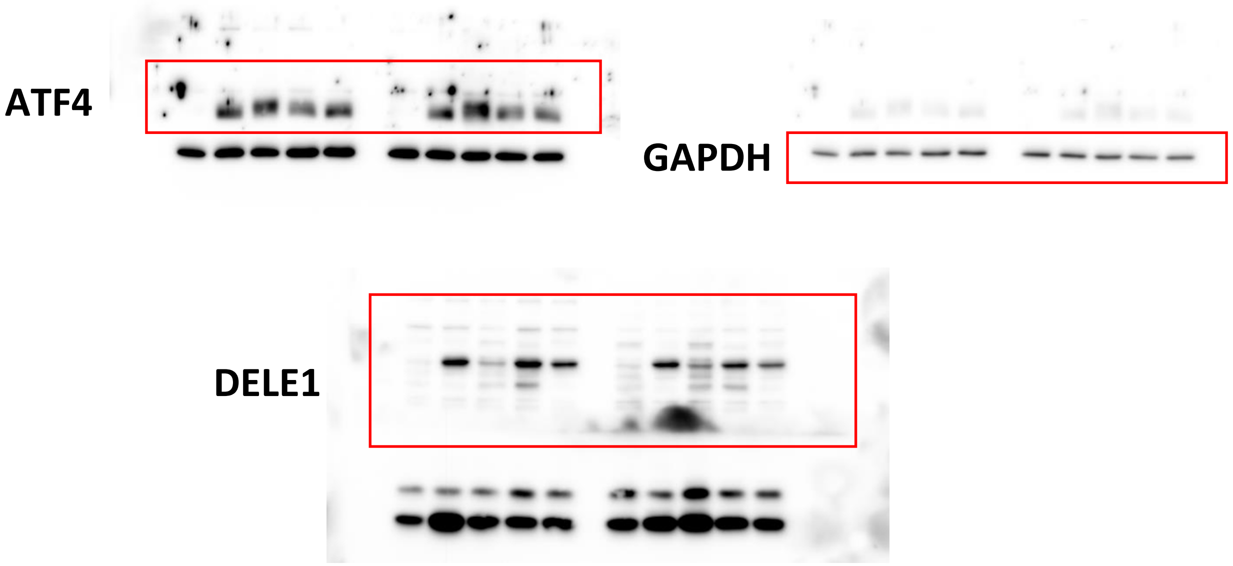

Supplementary Fig. 3e

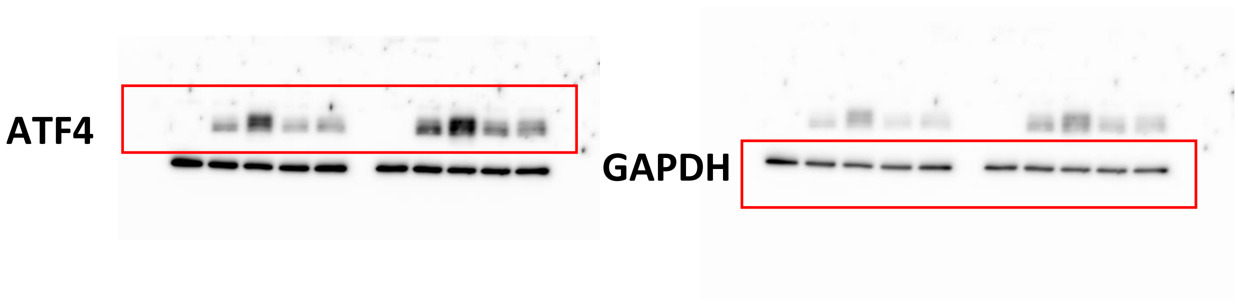

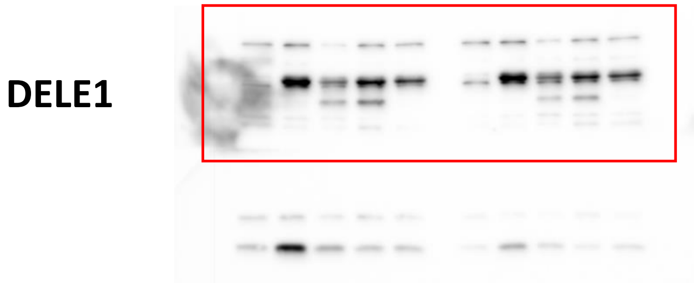

Supplementary Fig. 3f

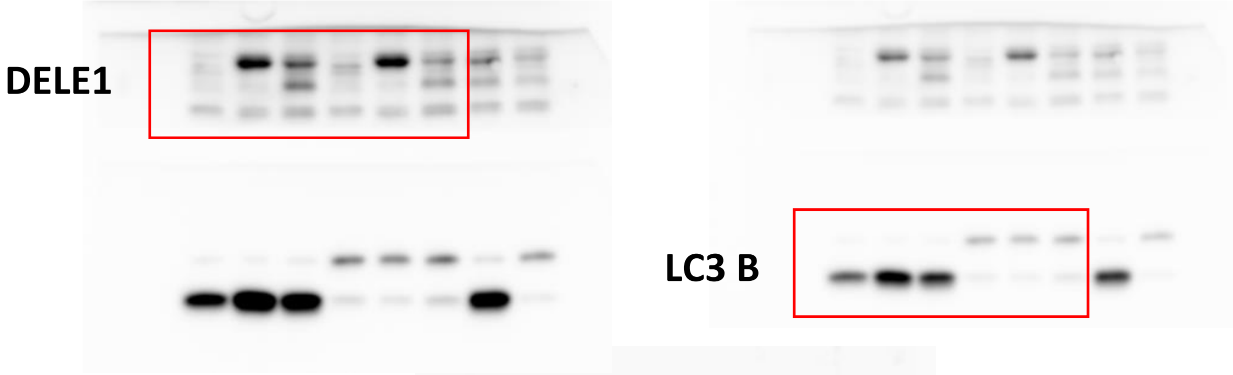

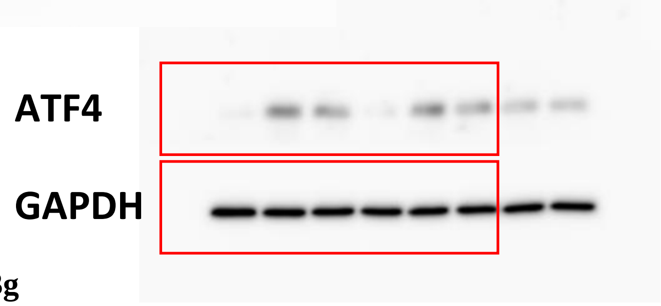

Supplementary Fig. 3g

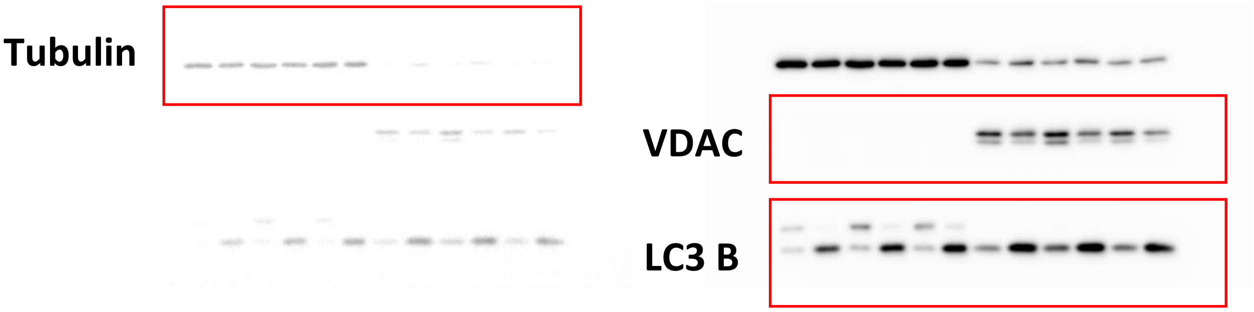

Supplementary Fig. 4a

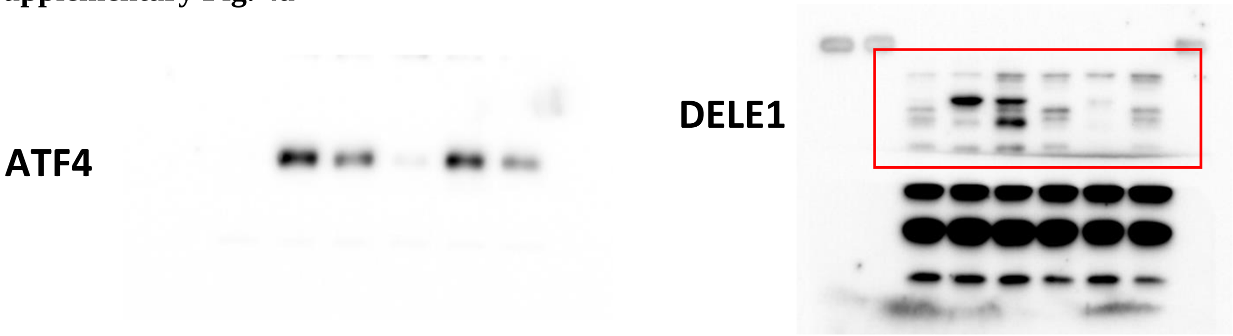

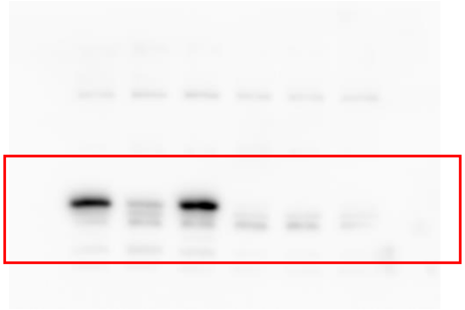

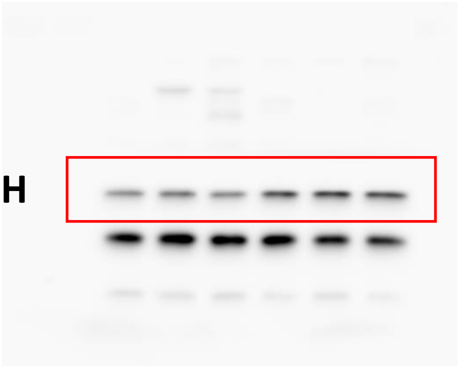

Supplementary Fig. 4b

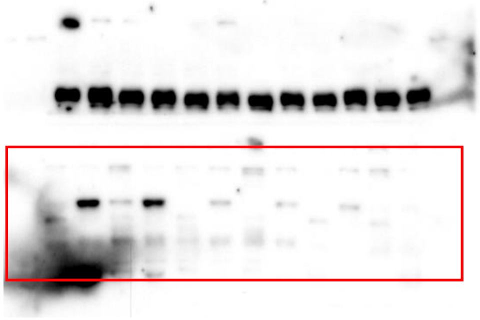

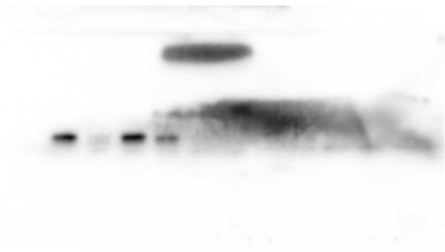

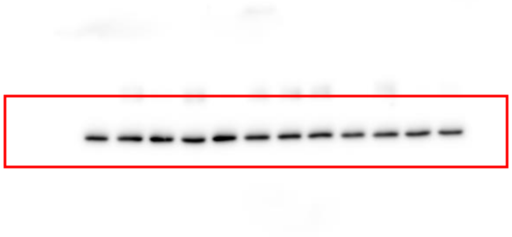

Supplementary Fig. 4c

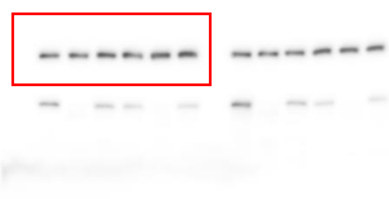

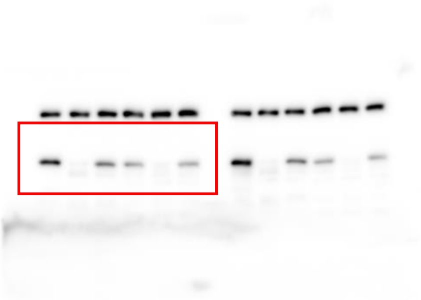

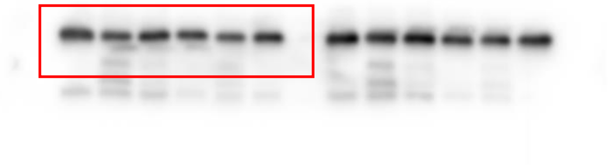

Supplementary Fig. 4d

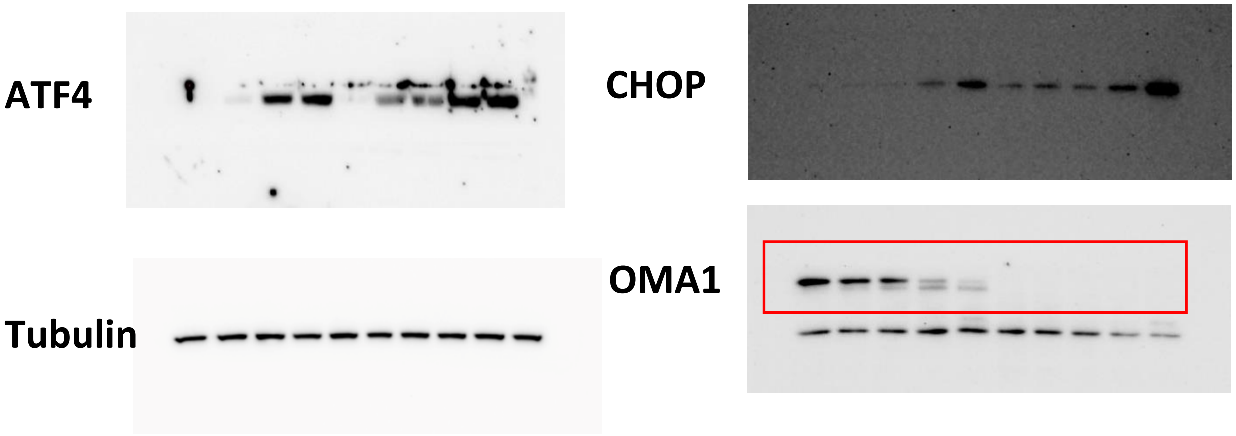

Supplementary Fig. 4e

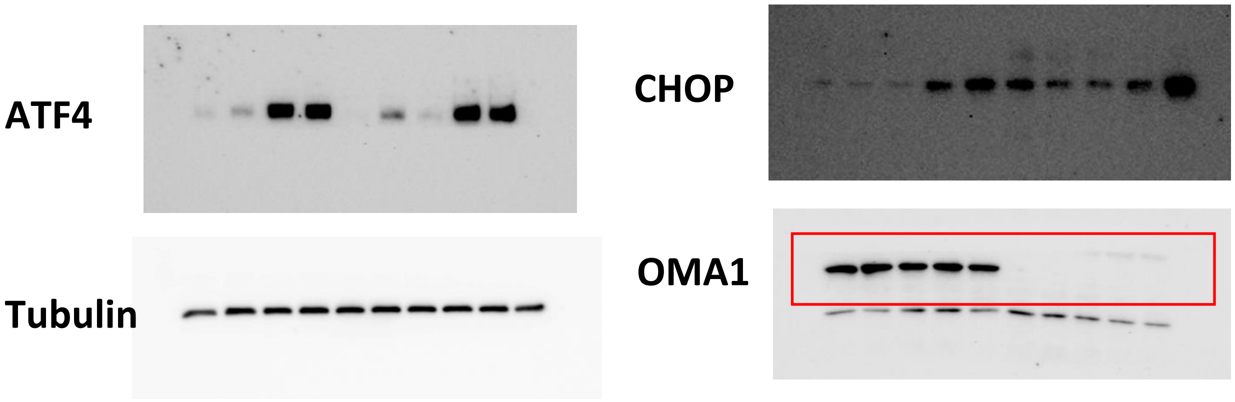

Supplementary Fig. 5a

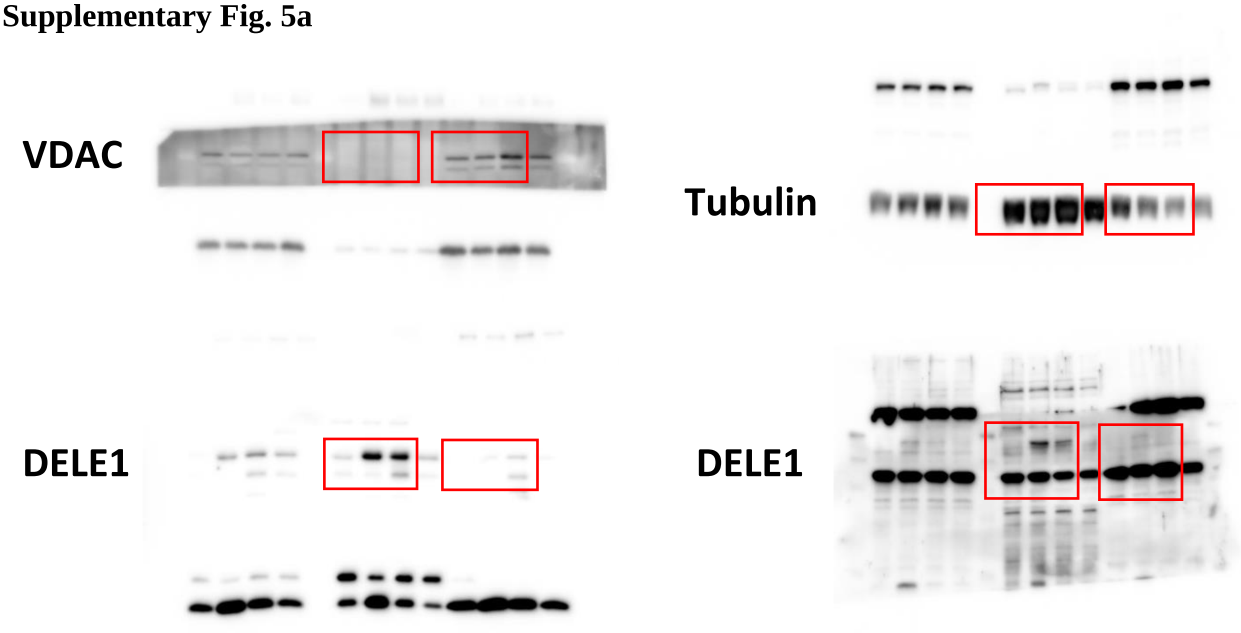

Supplementary Fig. 5b

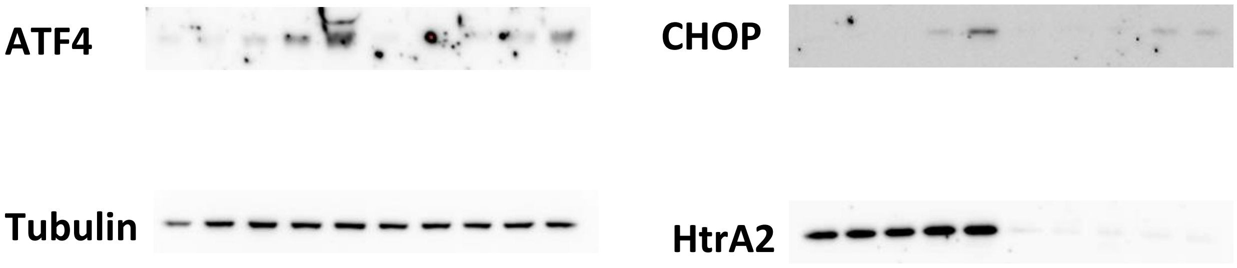

Supplementary Fig. 5c

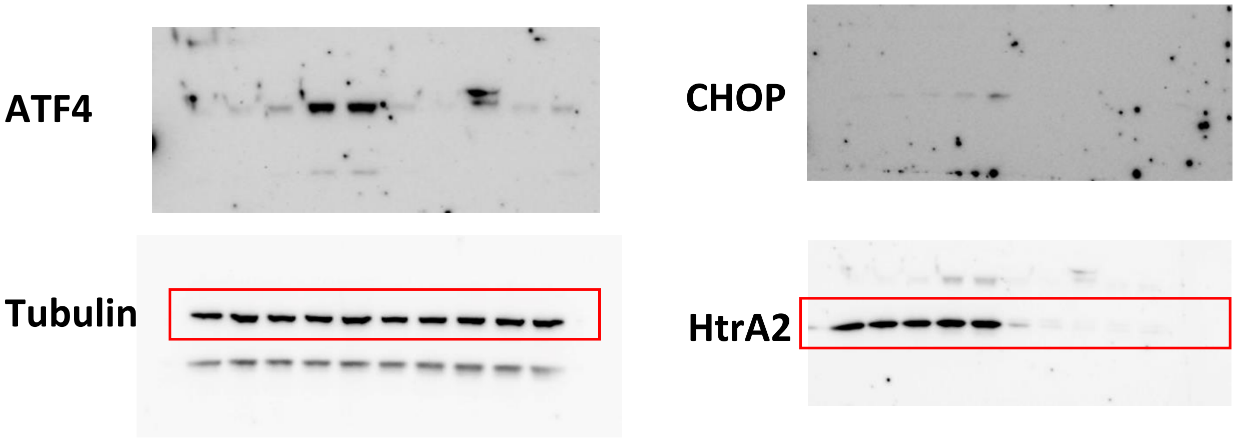

Supplementary Fig. 5d

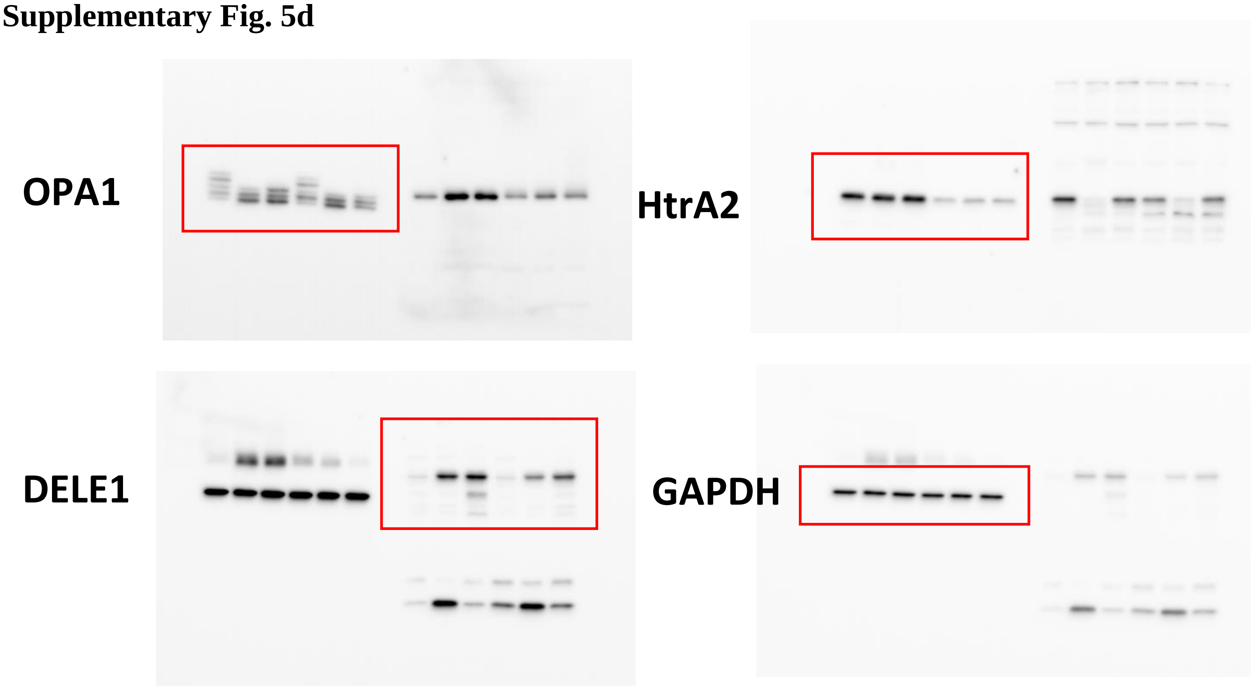

Supplementary Fig. 5e

ATF4

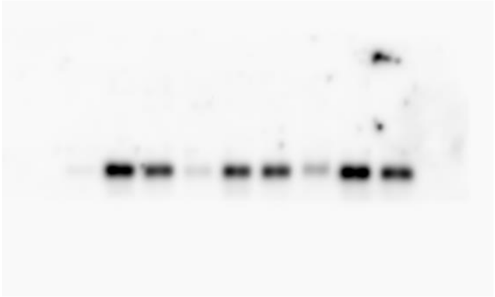

HtrA2

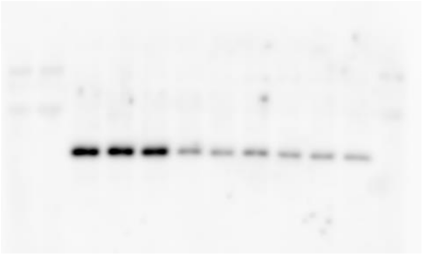

GAPDH

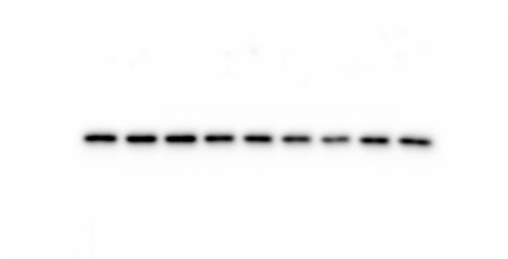

DELE1

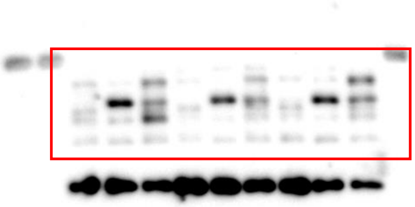

Supplementary Fig. 5f

Tubulin

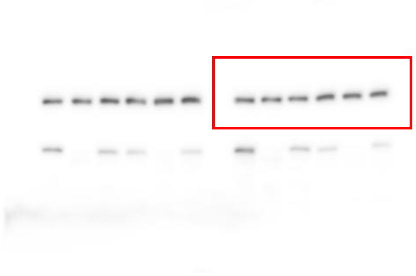

HtrA2

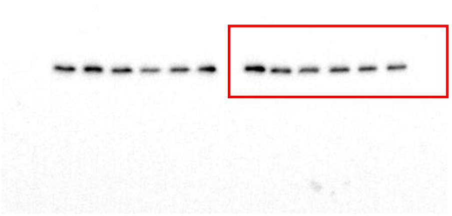

TIM23

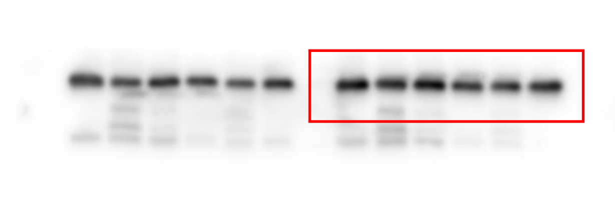

Supplementary Fig 6. Original uncropped blot and gel images of Fig. 1-6 and Supplementary Fig. 1-5.
