# Supplementary material for: Cytosolic retention of HtrA2 during mitochondrial protein import stress triggers the DELE1-HRI pathway
Source: Commun Biol. 2024 Mar 30;7:391. doi: 10.1038/s42003-024-06107-7 (PMC10981713; doi:10.1038/s42003-024-06107-7)
Supplement: Supplementary file 3 — Reporting Summary [file 42003_2024_6107_MOESM3_ESM.pdf]

nature portfolio

Corresponding author(s): Stephen Girardin

Last updated by author(s): May 30, 2023

Reporting Summary

Nature Portfolio wishes to improve the reproducibility of the work that we publish. This form provides structure for consistency and transparency in reporting. For further information on Nature Portfolio policies, see our Editorial Policies and the Editorial Policy Checklist.

Statistics

For all statistical analyses, confirm that the following items are present in the figure legend, table legend, main text, or Methods section.

n/a	Confirmed	
☒	☐	The exact sample size (*n*) for each experimental group/condition, given as a discrete number and unit of measurement
☒	☐	A statement on whether measurements were taken from distinct samples or whether the same sample was measured repeatedly
☒	☐	The statistical test(s) used AND whether they are one- or two-sided<br>*Only common tests should be described solely by name; describe more complex techniques in the Methods section.*
☒	☐	A description of all covariates tested
☒	☐	A description of any assumptions or corrections, such as tests of normality and adjustment for multiple comparisons
☒	☐	A full description of the statistical parameters including central tendency (e.g. means) or other basic estimates (e.g. regression coefficient) AND variation (e.g. standard deviation) or associated estimates of uncertainty (e.g. confidence intervals)
☒	☐	For null hypothesis testing, the test statistic (e.g. *F*, *t*, *r*) with confidence intervals, effect sizes, degrees of freedom and *P* value noted<br>*Give P values as exact values whenever suitable.*
☒	☐	For Bayesian analysis, information on the choice of priors and Markov chain Monte Carlo settings
☒	☐	For hierarchical and complex designs, identification of the appropriate level for tests and full reporting of outcomes
☒	☐	Estimates of effect sizes (e.g. Cohen's *d*, Pearson's *r*), indicating how they were calculated

Our web collection on statistics for biologists contains articles on many of the points above.

Software and code

Policy information about availability of computer code

Data collection	Western blot data were captured by GeneSys Version 1.7.2.27. Models were established by BioRender online.
Data analysis	N/A

For manuscripts utilizing custom algorithms or software that are central to the research but not yet described in published literature, software must be made available to editors and reviewers. We strongly encourage code deposition in a community repository (e.g. GitHub). See the Nature Portfolio guidelines for submitting code & software for further information.

Data

Policy information about availability of data

All manuscripts must include a data availability statement. This statement should provide the following information, where applicable:

- Accession codes, unique identifiers, or web links for publicly available datasets
- A description of any restrictions on data availability
- For clinical datasets or third party data, please ensure that the statement adheres to our policy

This study does not generate publicly available datasets. The data that supports the findings of this study are available in the article, figures and the supplementary figures. Uncropped and unedited blot images are provided in Supplementary Fig. 6.

Research involving human participants, their data, or biological material

Policy information about studies with human participants or human data. See also policy information about sex, gender (identity/presentation), and sexual orientation and race, ethnicity and racism.

Reporting on sex and gender	N/A
Reporting on race, ethnicity, or other socially relevant groupings	N/A
Population characteristics	N/A
Recruitment	N/A
Ethics oversight	N/A

Note that full information on the approval of the study protocol must also be provided in the manuscript.

Field-specific reporting

Please select the one below that is the best fit for your research. If you are not sure, read the appropriate sections before making your selection.

☒ Life sciences ☐ Behavioural & social sciences ☐ Ecological, evolutionary & environmental sciences

For a reference copy of the document with all sections, see nature.com/documents/nr-reporting-summary-flat.pdf

Life sciences study design

All studies must disclose on these points even when the disclosure is negative.

Sample size	Since this study does not contain animal or human subjects, there is no sample size evaluation involved. For cell culture experiments, individual cell culture well contains ~10 thousands to millions of cells based on the optimum confluence for experiments and common practice of the field.
Data exclusions	N/A
Replication	Experiments are independently repeated and the results are reproducible. The data shown in the article are representative of the number of individual experiments listed: Fig. 1a, n=5; Fig. 1b, n=2; Fig. 1c, n=3; Fig. 1d, n=3; Fig. 1e, n=3; Fig. 1f, n=3; Fig. 1g, n=3; Fig. 1h, n=2; Fig. 2a, n=2; Fig. 2b, n=2; Fig. 2c, n=2; Fig. 3a, n=2; Fig. 3c, n=3; Fig. 3d, n=3; Fig. 3e, n=3; Fig. 3h, n=2; Fig. 4a, n=3; Fig. 4b, n=3; Fig. 4c, n=3; Fig. 4d, n=3; Fig. 4e, n=3; Fig. 4f, n=3; Fig. 5a, n=3; Fig. 5b, n=3; Fig. 5c, n=3; Fig. 5d, n=3; Fig. 5e, n=3; Fig. 5f, n=3; Fig. 6a, n=2; Fig. 6b, n=3; Fig. 6c, n=3; Fig. 6d, n=3; Fig. 6e, n=2; Fig. 6f, n=2. Supplementary Fig. 1a, n=2; Supplementary Fig. 1a, n=2; Supplementary Fig. 1b, n=2; Supplementary Fig. 1c, n=3; Supplementary Fig. 1d, n=2; Supplementary Fig. 1e, n=2; Supplementary Fig. 1f, n=2; Supplementary Fig. 1g, n=2; Supplementary Fig. 1h, n=2; Supplementary Fig. 2, n=2; Supplementary Fig. 3a, n=2; Supplementary Fig. 3b, n=2; Supplementary Fig. 3c, n=3; Supplementary Fig. 3d, n=2; Supplementary Fig. 3e, n=3; Supplementary Fig. 3f, n=2; Supplementary Fig. 3g, n=3; Supplementary Fig. 4a, n=2; Supplementary Fig. 4b, n=2; Supplementary Fig. 4c, n=2; Supplementary Fig. 4d, n=2; Supplementary Fig. 4e, n=2; Supplementary Fig. 5a, n=2; Supplementary Fig. 5b, n=4; Supplementary Fig. 5c, n=4; Supplementary Fig. 5d, n=3, Supplementary Fig. 5e, n=2, Supplementary Fig. 5f, n=2.
Randomization	Randomization is not relevant to this study as all experiments are done in-vitro. Cells are cultured under identical conditions listed in the Method section, and are properly mixed before seeding in wells for experiments.
Blinding	Blinding is not relevant to this study as all experiments are done in-vitro.

Reporting for specific materials, systems and methods

We require information from authors about some types of materials, experimental systems and methods used in many studies. Here, indicate whether each material, system or method listed is relevant to your study. If you are not sure if a list item applies to your research, read the appropriate section before selecting a response.

Materials & experimental systems

n/a	Involved in the study
☐	☒ Antibodies
☐	☒ Eukaryotic cell lines
☒	☐ Palaeontology and archaeology
☒	☐ Animals and other organisms
☒	☐ Clinical data
☒	☐ Dual use research of concern
☒	☐ Plants

Methods

n/a	Involved in the study
☒	☐ ChIP-seq
☒	☐ Flow cytometry
☒	☐ MRI-based neuroimaging

Antibodies

Antibodies used

Antibodies used in this study are listed in the antibodies section of Methods.
ATF4 (11815S, Cell Signaling, 1/2000), DELE1(PA5-57712, Thermo Fisher Scientific, 1/2000), HRI (20499-1-AP, Proteintech, 1/3000), GAPDH (5174S, Cell Signaling, 1/10000), HtrA2 (#9745, Cell Signaling, 1/1000), OMA1 (HPA055120, Sigma, 1/2500), OPA1 (612606, BD, 1/2500), NLRX1 (MA5-27207, Thermo Fisher Scientific, 1/3000), RRBP1 (PA5-21392, Thermo Fisher Scientific, 1/2000), Tubulin (T9026, Millipore Sigma, 1/10,000), VDAC1 (ab14734, abcam, 1/2000), COX4 (ab33985, abcam, 1/4000), LC3B (3868S, Cell Signaling, 1/1000, PINK1 (BC100-494, Novus, 1/1000), TOMM20 (42406, Cell Signaling, 1/2000), TOM40 (18409-1-AP, Proteintech, 1/10000), TOM70 (14528-1-AP, Proteintech, 1/10000), TIMM22 (14927-1-AP, Proteintech, 1/2500), MIA40 (21090-1-AP, Proteintech, 1/2500), TIMM23 (11123-1-AP, Proteintech, 1:2000), HA Tag (ab18181, abcam, 1/1000), FLAG Tag (14793S, Cell Signaling, 1/1000), LONP1 (15440-1-AP, Proteintech, 1/1000), Myc-tag (2276S, Cell Signaling, 1/1000), HRP Rabbit (111-035-003, Jackson ImmunoResearch Laboratories, 1/10,000), HRP mouse (115-035-003, Jackson ImmunoResearch Laboratories, 1/10,000), Alexa fluor 488 rabbit (A11034, Life Technologies, 1/300), Cy3-conjugated goat anti-mouse (115-165-003, Jackson ImmunoResearch Laboratories, 1/300).

Validation

For Western blot, the predicted size and the actual size of the protein was analyzed by protein ladder. HA tag and FLAG tag antibodies are validated in over-expression studies. Additionally, for HRI, DELE1, OMA1, HtrA2, TOM20, TOM40, TOM70, TIM22, TIM23, MIA40, LONP1, NLRX1, RRBP1 and PINK1, these antibodies are tested by using siRNA or shRNA knockdown cells in this study.

Eukaryotic cell lines

Policy information about cell lines and Sex and Gender in Research

Cell line source(s)

HEK293T, HeLa, MEF and HCT116 cells are obtained from ATCC.

Authentication

Cell lines are authenticated by ATCC when purchased. Morphology of cells is monitored while the cells are growing.

Mycoplasma contamination

All cell lines tested negative for Mycoplasma contamination by PCR.

Commonly misidentified lines
(See ICLAC register)

No commonly misidentified lines are used in this study.
